# Optimizing genomic prediction for complex traits via investigating multiple factors in switchgrass

**DOI:** 10.1093/plphys/kiaf188

**Published:** 2025-05-07

**Authors:** Peipei Wang, Fanrui Meng, Christina Brady Del Azodi, Kenia Estefania Segura Abá, Michael D Casler, Shin-Han Shiu

**Affiliations:** DOE Great Lakes Bioenergy Research Center, Michigan State University, East Lansing, MI 48824, USA; Kunpeng Institute of Modern Agriculture at Foshan, Agricultural Genomics Institute at Shenzhen, Chinese Academy of Agricultural Sciences, Shenzhen, Guangdong 518124, China; Department of Plant Biology, Michigan State University, East Lansing, MI 48824, USA; DOE Great Lakes Bioenergy Research Center, Michigan State University, East Lansing, MI 48824, USA; Department of Plant Biology, Michigan State University, East Lansing, MI 48824, USA; Department of Plant Biology, Michigan State University, East Lansing, MI 48824, USA; DOE Great Lakes Bioenergy Research Center, Michigan State University, East Lansing, MI 48824, USA; Genetics and Genome Sciences Program, Michigan State University, East Lansing, MI 48824, USA; Department of Plant and Agroecosystem Sciences, University of Wisconsin, Madison, WI 53706, USA; DOE Great Lakes Bioenergy Research Center, Michigan State University, East Lansing, MI 48824, USA; Department of Plant Biology, Michigan State University, East Lansing, MI 48824, USA; Genetics and Genome Sciences Program, Michigan State University, East Lansing, MI 48824, USA; Department of Computational Mathematics, Science, and Engineering, Michigan State University, East Lansing, MI 48824, USA

## Abstract

Genomic prediction has accelerated breeding processes and provided mechanistic insights into the genetic bases of complex traits. To further optimize genomic prediction, we assess the impact of genome assemblies, genotyping approaches, variant types, allelic complexities, polyploidy levels, and population structures on the prediction of 20 complex traits in switchgrass (*Panicum virgatum* L.), a perennial biofuel feedstock. Surprisingly, short read-based genome assembly performs comparably to or even better than long read-based assembly. Due to higher gene coverage, exome capture and multi-allelic variants outperform genotyping-by-sequencing and bi-allelic variants, respectively. Tetraploid models show higher prediction accuracy than octoploid models for most traits, likely due to the greater genetic distances among tetraploids. Depending on the trait in question, different types of variants need to be integrated for optimal predictions. Our study provides insights into the factors influencing genomic prediction outcomes, guiding best practices for future studies and for improving agronomic traits in switchgrass and other species through selective breeding.

## Teaser

Exome capture and multi-allelic variants lead to better prediction for complex traits than genotyping-by-sequencing and bi-allelic variants due to higher gene coverage in switchgrass, respectively.

## Introduction

Before the genomic era, the breeding process relied heavily on natural variation, and hybridization or mutagenesis, and was time- and labor-consuming. In the genomic era, the molecular variant-assisted breeding technologies, especially genomic selection or genomic prediction (GP), have dramatically accelerated the breeding processes for both animals and plants (Crossa et al. 2017; Alemu et al. 2024; Tade and Melesse 2024). Learning from a training population with both genetic variants (e.g. single nucleotide polymorphisms [SNPs]) and phenotypic data (e.g. flowering time), GP establishes statistical models to build the connection between the genetic variants and phenotypic data, which can be applied to a new population that has been genotyped but with unmeasured phenotypes. This can be done without planting all the seedlings and waiting for a whole growing season, therefore resulting in shortened breeding cycles (Estopa et al. 2023; Alemu et al. 2024).

Substantial effort has been made to understand the effects of different factors on the accuracy of GP (Norman et al. 2018; Zhang et al. 2019; Kriaridou et al. 2020; Batista et al. 2022; Alemu et al. 2024; Zheng et al. 2024), including variant density (Kriaridou et al. 2020), population size (Akdemir et al. 2019; Wu et al. 2023), population structure (Norman et al. 2018), relationship between training and test populations (Isidro et al. 2015), functional genomic annotations (Zheng et al. 2024), marker ploidy (Aalborg and Nielsen 2024), variants besides SNPs (e.g. structural variants, Liu et al. 2024), and statistical methods (Azodi et al. 2019; Zhang et al. 2019; Wang et al. 2023b; Alemu et al. 2024). For example, the prediction accuracy increases along with the increase of variant density to a certain point (Norman et al. 2018; Kriaridou et al. 2020). Regarding the statistical methods used in GP, a relatively simple linear model, ridge regression Best Linear Unbiased Prediction (rrBLUP), performed similarly or better compared with other “state-of-the-art” approaches for most scenarios, such as random forest, support vector machine, gradient boosting, artificial neural net, and convolutional neural net (Azodi et al. 2019; Azodi et al. 2020; Wang et al. 2024). Beyond the above, there are additional factors with their effects on GP accuracy remaining largely unexplored, such as genome assemblies, genotyping approaches, and variant allelic complexities. With reduced sequencing cost and improvement in assembly algorithms, a number of plant species have genome assemblies improved from scaffold-level to chromosome-level or even telomere-to-telomere (Chen et al. 2023; Lu et al. 2024). However, the higher the quality of the assembly, the more the cost needed, and it remains unknown whether a better assembly is necessarily to improve the GP accuracy since few studies have focused on the effects of assembly quality on GP accuracy. A blueberry (*Vaccinium corymbosum*) study showed that the chromosome-level assembly “Draper” led to comparable prediction accuracy for most traits as the scaffold-level assembly “W8520” with only half numbers of probes (Benevenuto et al. 2019), but it was still unknown whether the chromosome-level assembly led to improved GP accuracy or not when the genome-wide variants were used.

A number of approaches have been developed for genotyping, including SNP array, genotyping by random amplicon sequencing-direct system, genotyping-by-sequencing (GBS), exome capture (EC), whole genome sequencing (WGS), and others (Scheben et al. 2017; Ayalew et al. 2022; Boatwright et al. 2022; Wang et al. 2023a; Minamikawa et al. 2024). WGS covers the majority of genetic variants across the whole genome, whereas the other approaches only cover a portion of variants but with relatively lower costs. It remains unclear how different genotyping approaches impact the GP accuracy and which approaches would be a better option with different aims of the studies.

The genetic variants can be both bi-allelic and multi-allelic: out of the SNPs called for the Arabidopsis 1001G data using the 250k SNP-array from Kim et al. (2007), 7% were multi-allelic (Alonso-Blanco et al. 2016); out of the SVs identified using 32 *A. thaliana* ecotypes, multi-allelic SVs (29.65 mb) were much more than bi-allelic SVs (7.51 mb, Kang et al. 2023). However, the majority of past genome-wide association study (GWAS) and GP studies mainly used bi-allelic variants (Crossa et al. 2017; Misra et al. 2017; Song et al. 2018; Wang et al. 2023b). It is important to know how the multi-allelic variants contribute to complex trait prediction to make the best of the ever increasing genomic data, such as graph-based pan-genomes that are used as references to call all types of genetic variants with high quality.

Here, using switchgrass (*Panicum virgatum* L.)—a perennial grass with both tetra- and octo-ploids and a key species as a bioenergy feedstock (McLaughlin and Adams Kszos 2005)—as a model system, we aim to optimize GP by assessing the impacts on GP accuracy due to differences in (i) genome assembly versions, (ii) genotyping strategies, (iii) genetic variant types, (iv) numbers of variant alleles, and (v) polyploids and population structures. Both short read and long read-based assemblies are available for switchgrass, and there are 2 genotyping data sets for a switchgrass diversity panel with 486 individuals: GBS (Lu et al. 2013) and EC (Evans et al. 2015). We also assess how different types of genetic variants impact our ability to uncover the genetic basis underlying anthesis date by interpreting GP models.

## Results

### Baseline models predicted trait values with variable accuracy

**Figure 1.**
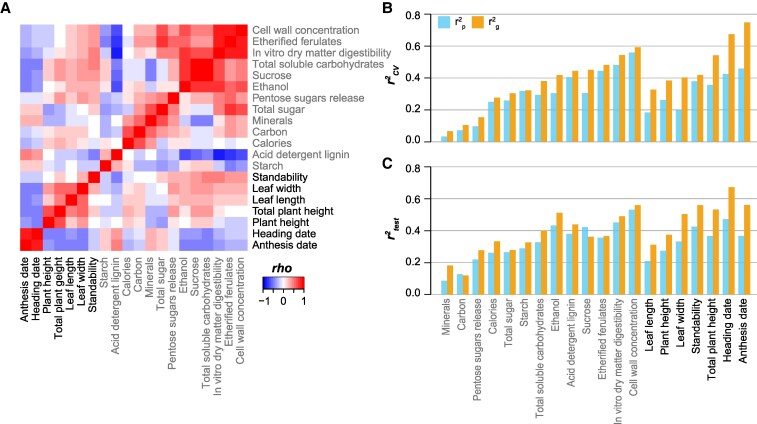
Correlation between the values of 20 traits and the prediction accuracy for these 20 traits. **A)** Heatmap showing the correlation between the values of 20 traits. Gray font: 13 biochemical traits; black font: 7 morphological traits. *rho*: Spearman's rank correlation coefficient. **B** and **C)** Prediction accuracy (*r*^2^) for 20 traits in CV **B)** and test **C)** sets for models built using the bi-allelic SNPs called by mapping the GBS data to v5 genome assembly. The models were built on all individuals in the training set including tetraploids and octoploids. *x* axis: 20 traits; *y* axis: median *r*^2^ between true and predicted trait values among 10 replicate runs. *r*^2^_p_: *r*^2^ for models built with the population structure, which was defined as the first 5 principal components from the genetic variants; *r*^2^_g_: *r*^2^ of models built with genetic variants.

There are 20 phenotypic trait values available for the switchgrass diversity panel we used (Supplementary Table S1), including 7 morphological (anthesis date, heading date, plant height, total plant height, leaf length, leaf width, and standability) and 13 biochemical traits (starch, acid detergent lignin, calories, carbon, minerals [total ash], total sugar, pentose sugars release/g dry forage, ethanol/g dry forage, sucrose, total soluble carbohydrates, in vitro dry matter digestibility, etherified ferulates, and cell wall concentration) (Supplementary Table S2, Lipka et al. 2014). Among the 20 traits, some are highly associated with each other, such as 3 biochemical traits, namely, cell wall concentration, etherified ferulates, and in vitro dry matter digestibility, and 2 flowering time-related traits, heading date and anthesis date (Fig. 1A). To determine how well these 20 phenotypic traits can be predicted in switchgrass, we first built GP models using GBS bi-allelic SNPs that were mapped to the version 5 genome assembly (v5, long read-based) of the lowland tetraploid AP13 (Lovell et al. 2021). The rrBLUP approach was used to build the models due to its relatively better or similar prediction accuracy compared with other methods and its interpretability for underlying molecular mechanisms for target traits (Azodi et al. 2019, 2020; Wang et al. 2024). Information of 20% of the individuals in the diversity panel was held out as the test set before model building to serve as an independent data set for evaluating the performance of final models. The data of the remaining 80% individuals were used to train the models with a 5-fold cross-validation (CV) scheme (see Materials and methods). To compare prediction accuracy among different traits, we also established models using the population structure (p; defined as the first 5 principal components of the corresponding genetic variants [g], see Materials and methods) to determine the baseline prediction accuracy for each trait (Azodi et al. 2020). The *r*^2^ of the Pearson correlation coefficient (PCC) between the true and predicted trait values for individuals was first calculated for models built using genetic variants (*r*^2^_g_) and population structure (*r*^2^_p_), separately. Then, the improvement in *r*^2^ (*r*^2^_i_ = *r*^2^_g_ − *r*^2^_p_) was used to measure the performance of the GP models. This *r*^2^_i_ was calculated both for individuals in the CV set (r^2^_i,CV_) and the test set (*r*^2^_i,test_).

We found that *r*^2^_i,CV_ was positively correlated with *r*^2^_i,test_ (PCC = 0.68, *P*-value = 9.89e-04; Fig. 1, B and C), suggesting the relative robustness of our models. In general, *r*^2^_i,CV_ for morphological traits (average *r*^2^_i,CV_ = 0.176) were higher than those for biochemical traits (average *r*^2^_i,CV_ = 0.055, *P*-value of Wilcoxon signed-rank test = 4.66e-03), and the 2 flowering time-related traits—anthesis date and heading date—had the highest *r*^2^_i,CV_ (0.289 and 0.250, respectively). These results indicate that morphological traits tended to have higher heritability, whereas the prediction accuracy (*r*^2^_g_) for most biochemical traits was mainly confounded by population structure. Thus, cautions need to be taken in the GWAS and GP practices for those biochemical traits.

### Better genome assembly did not provide better trait prediction

**Figure 2.**
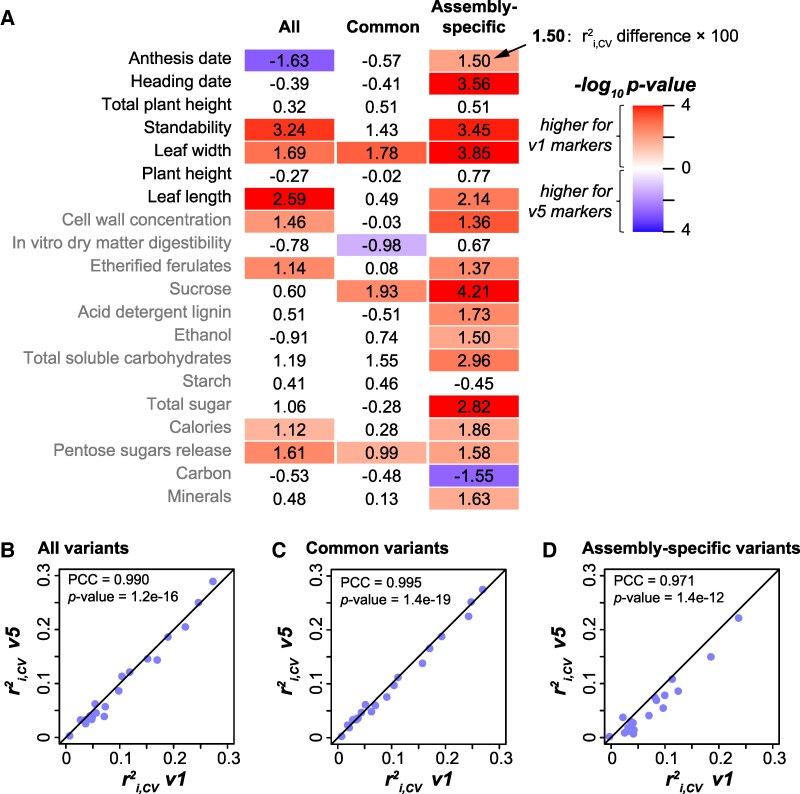
Prediction accuracy differences between models based on v1 and v5 assemblies. **A)** Differences in *r*^2^_i,CV_ between v1- and v5-based models. The column “All”: models built using all the v1- and v5-based GBS bi-allelic SNPs; “Common”: models built using the common GBS bi-allelic SNPs shared between v1 and v5 assemblies; “Assembly-specific”: models built using GBS bi-allelic SNPs that are specific to v1 and v5 assemblies, separately. Values in the heatmap: *r*^2^_i,CV_ differences × 100; only the comparisons with statistical significance (*P* from Wilcoxon signed-rank test < 0.05) are indicated with colors. **B** to **D)** Correlation between *r*^2^_i,CV_ of v1- (*x* axis) and v5-based (*y* axis) models. **B)** Models built using all the v1- and v5-based GBS bi-allelic SNPs. **C)** Models built using the common GBS bi-allelic SNPs shared between v1 and v5 assemblies. **D)** Models built using GBS bi-allelic SNPs that are specific to v1 and v5 assemblies, separately.

To test whether the genome assembly with improved quality (i.e. v5, Lovell et al. 2021) would lead to higher prediction accuracy for switchgrass traits than short read-based assembly (e.g. v1), we also mapped GBS sequences to the v1 assembly and called the genetic variants using the same methods and parameter settings as those for v5-based variants (see Materials and methods). The assembly sizes of v1 (1,230 mb) and v5 assemblies (1,129 mb) were similar, and there were almost equivalent numbers of variants for v1 (11,042) and v5 (11,020) assemblies, with similar distribution of variants across different gene functional regions (Supplementary Fig. S1, A and B, and Table S3). In addition, most of the GBS bi-allelic SNPs were shared between these 2 assemblies, as 5,283 (69.92%) out of 7,556 v1-based bi-allelic SNPs had corresponding v5-based ones (see Materials and methods and Supplementary Table S4). Beyond these similarities, N50 for v5 assembly (N50 = 5.5 mb) is nearly 100 times higher than v1 (N50 = 54 kb). However, v5-based models outperformed v1-based models only for anthesis date, with the average difference in *r*^2^_i,CV_ between 2 models for 20 traits was only 0.006 (the “All” column in Fig. 2, A and B; for the *r*^2^_p,CV_, *r*^2^_g,CV_, *r*^2^_i,CV_, *r*^2^_p,test_, *r*^2^_g,test_, and *r*^2^_i,test_, see Supplementary Figs. S2 to S7, respectively). This result suggests that the improvement in contig size may not be relevant to GP because genetic variants are treated as independent variables in GP algorithms.

In fact, although the effect sizes (differences in *r*^2^_i,CV_) were small, models built with variants derived from v1 had significantly better *r*^2^_i,CV_ than v5-based models for 7 traits (the “All” column in Fig. 2A). These differences in *r*^2^_i,CV_ can be partially explained by the assembly-specific variants: models built using common variants (shared between v1 and v5 assemblies) had similar performance (the “Common” column in Fig. 2, A and C), while assembly-specific variant-based models tended to have better performance when they were v1-based (the “Assembly-specific” column in Fig. 2, A and D). A possible explanation of this finding that needs to be investigated further is that, while v5 assembly had improved contig sizes, some genomic regions present in the v1 assembly were not assembled from long reads and thus were no longer present in the v5 assembly. These results suggest that, in switchgrass, short read-based assembly is equally competent as or even superior to long read-based assembly for the purpose of GP. Nevertheless, since the v5 assembly is used by the community, our subsequent analyses are based on genetic variants called using the v5 assembly.

### EC SNPs led to models better than those based on GBS

**Figure 3.**
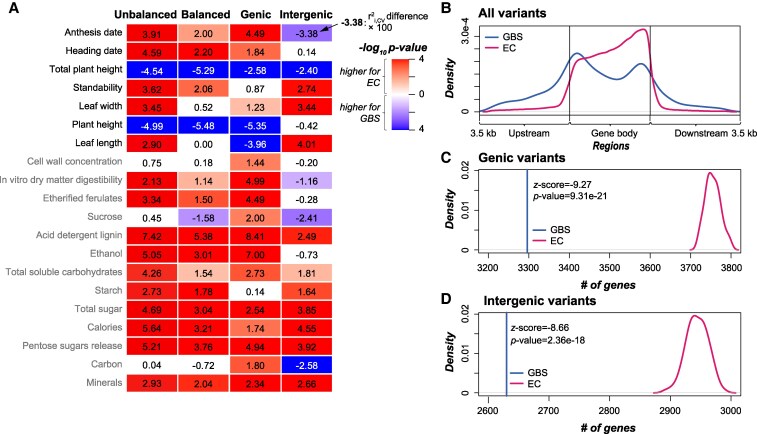
Better trait prediction in EC-based models than GBS-based models. **A)** Differences in *r*^2^_i,CV_ between EC- and GBS-based models. The column “Unbalanced”: models built using all EC and GBS bi-allelic SNPs; “Balanced”: models built using balanced EC (down-sampled to match the number of GBS variants) and GBS bi-allelic SNPs; “Genic”: models built using balanced EC and GBS bi-allelic SNPs that were located within the gene bodies; “Intergenic”: models built using balanced EC and GBS bi-allelic SNPs that were located within intergenic regions. Values in the heatmap: *r*^2^_i,CV_ differences × 100; only the comparisons with statistical significance (*P* from Wilcoxon signed-rank test < 0.05) are indicated with colors. **B)** Distribution of the GBS and EC bi-allelic SNPs in the genome. All the gene bodies are scaled to 3.5 kb to make the locations of variants within the gene body comparable. The regions of 3.5 kb upstream and downstream the gene bodies are also shown. **C** and **D)** Comparison between the numbers of genes harboring **C)** or adjacent to **D)** GBS bi-allelic SNPs and the down-sampled EC bi-allelic SNPs. *z*-score values (and the corresponding *P*-values) of the GBS-based numbers in the distribution of EC-based numbers are shown in the figures.

EC and GBS are 2 commonly used genotyping methods to capture the genetic variants with a much lower cost than WGS, but use different strategies: EC identifies genetic variants that may alter protein sequences, while GBS captures variants around restriction sites of (a) given restriction enzyme(s). We found that when using bi-allelic SNPs, EC-based models had significantly higher *r*^2^_i,CV_ than GBS-based models for 15 traits but lower *r*^2^_i,CV_ for only 2 traits (the “Unbalanced” column in Fig. 3A). Considering that there were ∼72 times more EC variants (526,705; Supplementary Fig. S1C) than GBS ones (7,357), to eliminate the influence of variant numbers on prediction accuracy, we randomly down-sampled EC bi-allelic SNPs to the same number of GBS bi-allelic SNPs and conducted this down-sampling 100 times (Materials and methods). The median *r*^2^_i,CV_ of the 100 models built with these down-sampled EC variants still had significantly higher r^2^_i,CV_ than GBS-based models for 13 traits, but had significantly lower *r*^2^_i,CV_ than GBS-based models for only 3 traits (the “Balanced” column in Fig. 3A). These results suggest that, regardless of the numbers of variants, the distribution of variants may be the main factor influencing GP accuracy when comparing EC- and GBS-based models.

We found that EC variants tended to be located in genic regions or in intergenic regions that are closer to genes (Fig. 3B; Supplementary Fig. S1) and contained a higher proportion of genic variants (86.4%) than GBS variants (55.5%; Supplementary Table S3). We further established GP models using only genic GBS and EC variants (down-sampled to match the number of genic GBS variants) and found that genic GBS-based models still had significantly lower *r*^2^_i,CV_ than genic EC-based models for 15 of 20 traits (the “Genic” column in Fig. 3A). When examining the GBS bi-allelic SNPs and the 100 down-sampled subsets of EC bi-allelic SNPs in detail, we found significantly fewer genes that contained GBS variants (3,296) than contained EC variants (median across 100 replicates = 3,754; *P*-value = 9.31e-21; Fig. 3C). These findings indicate that having higher gene coverage, rather than more genic variants, may explain the higher GP accuracy of EC-based models.

Furthermore, we also built models using intergenic GBS and EC bi-allelic SNPs and found that GBS-based models had significantly smaller *r*^2^_i,CV_ than EC-based models (balanced for variant numbers) for 10 of 20 traits, but significantly higher *r*^2^_i,CV_ only for 5 traits (the “Intergenic” column in Fig. 3A). Similar to genic variants, there were fewer genes adjacent to (3.5 kb up- or downstream the variants) intergenic GBS variants (2,630, *P*-value = 2.36e-18; Fig. 3D) than intergenic EC variants (down-sampled to match intergenic GBS, the median number of genes among 100 replicates was 2,944). This result suggests that, beyond higher gene coverage, including variants that are adjacent to more genes may also improve the GP accuracy, which is potentially due to the linkage between these intergenic variants and genes. Another potential explanation for the better performance of models built using intergenic EC variants was because the intergenic EC variants were closer to gene bodies than the intergenic GBS variants (Fig. 3B). If this was true, we would expect negative correlations between the distance to the gene body and the absolute coefficient of variants that was used as a proxy for the contribution of variants to trait prediction. However, we did not see such correlation for leaf width models as an example (Spearman's rank correlation coefficients = −7.7e-03 and 7.2e-03 for models built with intergenic GBS and EC bi-allelic SNPs, respectively), for which the EC-based model had significantly higher *r*^2^_i,CV_ than the GBS-based model (the “Intergenic” column in Fig. 3A). Taken together, those results suggest that EC variants are superior to GBS variants in predicting switchgrass traits, and gene coverage by variants has a substantial impact on how well a trait can be predicted.

### Multi-allelic variants outperformed bi-allelic variants in trait prediction

**Figure 4.**
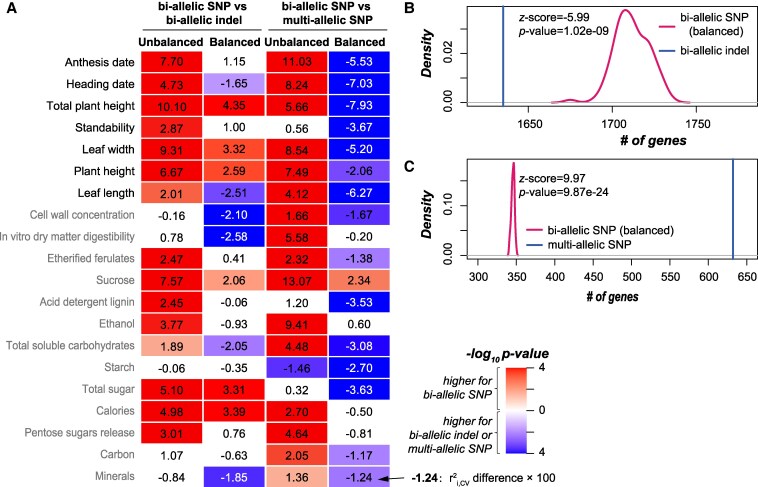
Performance of models built using bi-allelic indels and multi-allelic SNPs. **A)** Differences in *r*^2^_i,CV_ between models built using GBS bi-allelic SNPs and models built using GBS bi-allelic indels (left 2 columns) and between GBS bi-allelic SNP-based models and GBS multi-allelic SNP-based models (right 2 columns). The “Unbalanced” and “balanced” columns: the bi-allelic SNPs were not down-sampled and down-sampled to the same numbers of bi-allelic indels or multi-allelic SNPs, respectively. Values in the heatmap: *r*^2^_i,CV_ differences × 100; only the comparisons with statistical significance (*P* from Wilcoxon signed-rank test < 0.05) are indicated with colors. **B)** Comparison between the numbers of genes harboring or adjacent to the down-sampled bi-allelic SNPs and bi-allelic indels. **C)** Comparison between the numbers of genes harboring or adjacent to the down-sampled bi-allelic SNPs and multi-allelic SNPs. *z*-score values (and the corresponding *P*-values) of the bi-allelic indel-based or multi-allelic SNP-based numbers in the distribution of down-sampled bi-allelic SNP-based numbers are shown in the figures.

Up to this point, all the genetic variants we have explored are bi-allelic SNPs, which are commonly used in GWAS and GP studies. Besides bi-allelic SNPs, bi-allelic insertion–deletions (indels) and multi-allelic variants also contribute to phenotypic variation and are informative for trait prediction (Veerkamp et al. 2016; Biová et al. 2024), but with the degree to which they contribute to GP accuracy largely unexplored. For the GBS data, compared with the 7,357 bi-allelic SNPs, 1,827 bi-allelic indels were identified (Supplementary Table S3). Models built using bi-allelic indels had significantly lower *r*^2^_i,CV_ than SNP-based models for 15 traits (without down-sampling; the 1st column in Fig. 4A), but had comparable performance as balanced SNP-based models (with lower *r*^2^_i,CV_ for 6 traits and higher r^2^_i,CV_ for another 6 traits; the 2nd column in Fig. 4A). These results suggest that, although occurring less frequently than SNPs, indels are comparably useful as SNPs when the numbers are balanced.

In addition to bi-allelic indels, there were 1,836 multi-allelic variants identified for the GBS data, including multi-allelic SNPs (350), multi-allelic indels (604), and multi-allelic SNPs/indels (882) (before encoding; Supplementary Table S3). To simplify the comparison, we built models using only the 350 GBS multi-allelic SNPs. Multi-allelic SNP-based models had significantly lower *r*^2^_i,CV_ than models based on bi-allelic SNPs (without down-sampling) for 16 traits (the 3rd column in Fig. 4A), but had significantly higher *r*^2^_i,CV_ for 15 traits than models built using down-sampled bi-allelic SNPs (the 4th column in Fig. 4A). Consistent with our earlier results indicating the importance of gene coverage by variants in GP, we found 0.83 times more genes covered by multi-allelic SNPs (632) than balanced bi-allelic SNPs (median: 346, *P*-value = 9.87e-24; Fig. 4C), but slightly fewer genes covered by bi-allelic indels (1,635, compared with median 1,711 genes covered by the corresponding balanced bi-allelic SNPs, *P*-value = 1.02e-09; Fig. 4B). Taken together, these results indicate that multi-allelic SNPs outperform the commonly used bi-allelic SNPs in trait prediction, particularly in their contribution to improve the coverage of genes, and should be included in future studies.

**Figure 5.**
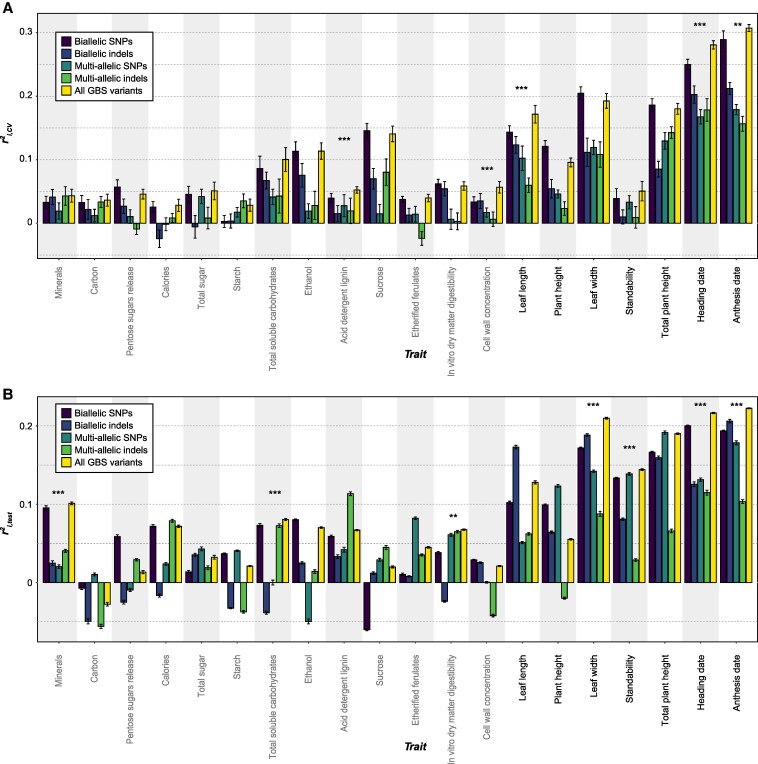
Improvement in prediction accuracy by integrating different types of GBS variants. **A** and **B)** The *r*^2^_i,CV_  **A)** and *r*^2^_i,test_  **B)** for models built using GBS bi-allelic SNPs, bi-allelic indels, multi-allelic SNPs, multi-allelic indels, and all the GBS variants. Column with ** or ***: the integrating model had significantly improved prediction accuracy compared with all the models built using a single type of GBS variants; **P-value from Wilcoxon signed-rank test < 0.01; ***P-value < 0.001. Error bar: standard deviation of 10 replicate runs.

Next, we asked whether the prediction can be improved by taking advantage of all the types of variants. We built integrated GP models using all the GBS variants, including 13,265 bi- and multi-allelic SNPs and indels in total. This was not conducted for EC variants, due to the large number of EC variants (2,552,214 variants in total) and the extremely high computing resource requirement to establish models using all the EC variants. We found that 5 and 7 traits had significantly improved *r*^2^_i,CV_ and *r*^2^_i,test_ in the integrated models compared with models that were built using individual types of GBS variants separately, respectively (Fig. 5; for *r*^2^_p_ and *r*^2^_g_, see Supplementary Fig. S8). These results suggest that different types of genetic variants should be integrated to improve the GP accuracy for switchgrass traits and potentially traits in other plant species as well.

### Models built for octoploids had lower trait prediction accuracy than those for tetraploids

**Figure 6.**
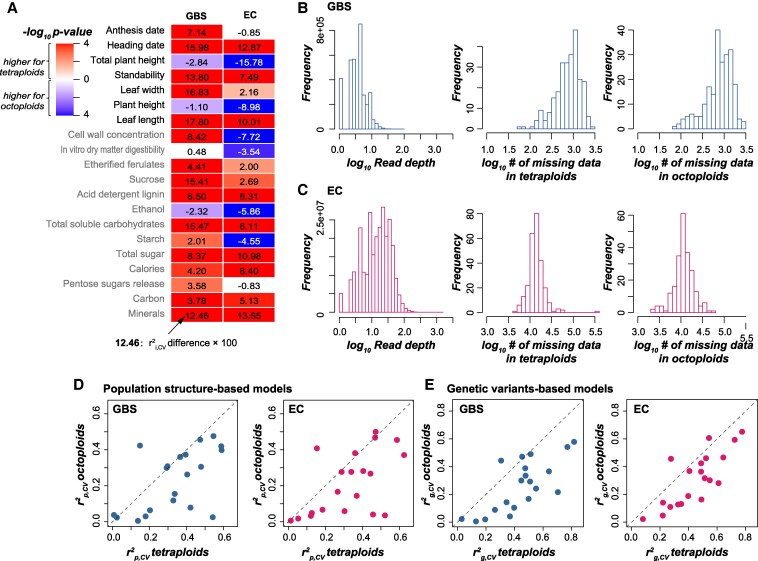
Models for octoploids had poorer prediction accuracy than models for tetraploids. **A)** Differences in prediction accuracy between models for tetra- and octoploids when GBS and EC bi-allelic SNPs were used. Values in the heatmap: *r*^2^_i,CV_ differences × 100; only the comparisons with statistical significance (*P* from Wilcoxon signed-rank test < 0.05) are indicated with colors. **B)** Read depth (left panel) and the number of missing data (middle and right panels) of the GBS data. **C)** Read depth (left panel) and the number of missing data (middle and right panels) of the EC data. **D** and **E**) Comparison of the *r*^2^_p,CV_  **D)** and *r*^2^_g,CV_  **E)** between models for tetraploids (*x* axis) and octoploids (*y* axis) when GBS (left panel) or EC (right panel) bi-allelic SNPs were used.

Thus far, the GP models were built for all individuals, including both tetraploids and octoploids, where the variants for octoploid individuals were treated using a pseudo-diploid genotyping strategy (see Materials and methods), which is commonly used to handle variants for individuals with different ploidy levels in previous studies. Considering the relatively higher complexity of variants for octoploid individuals, we asked whether the same variant set (GBS or EC) would have different prediction accuracy for tetraploid and octoploid individuals. When GBS bi-allelic SNPs were used, models built for tetraploids had significantly higher *r*^2^_i,CV_ than octoploid models for 16 traits, but significantly lower *r*^2^_i,CV_ for only 3 traits (the “GBS” column in Fig. 6A). One possible explanation of the lower *r*^2^_i,CV_ for octoploid models is that the variants called for octoploids were not as good as those for tetraploids in GBS data, due to the relatively low read depth (RD) of GBS bi-allelic SNPs (average at 4.41; left panel in Fig. 6B), and potentially a need for deeper RD for octoploids than tetraploids in variant calling. In contrast, the EC data had an average RD at 21.10 (left panel in Fig. 6C), and much lower proportions of missing data for both tetraploids (3.1% [16,237], middle panel in Fig. 6C, compared with 12.5% [916] for GBS; Fig. 6B) and octoploids (2.4% [13,023], right panel in Fig. 6C, compared with 12.1% [893] for GBS; Fig. 6B). EC-based models for tetraploids still had significantly higher *r*^2^_i,CV_ than octoploid models for 12 traits (for the GBS-based models, this number was 16), but significantly lower *r*^2^_i,CV_ for the other 6 traits (3 for the GBS-based models) (the “Exome capture” column in Fig. 6A). These results indicate that the low RD in GBS data might, but only partially, explain the lower GP accuracy of octoploid models.

Another potential explanation for lower GP accuracy for octoploids is the different population structures between octoploids and tetraploids, since both *r*^2^_p,CV_ (Fig. 6D) and *r*^2^_g,CV_ (Fig. 6E) for octoploids were generally smaller than those values for tetraploids, regardless of whether GBS or EC data were used. One previous study (Evans et al. 2015) showed that the within-subpopulation genetic distances in 2 octoploid subpopulations were shorter than those in 3 tetraploid subpopulations (see Fig. 4 in Evans et al. 2015). We further built a model for each of 4 subpopulations with ≥85 individuals each (Supplementary Table S1) using EC bi-allelic SNPs and found that subpopulations with larger within-subpopulation genetic distances tended to have higher GP accuracy (either *r*^2^_p,CV_, *r*^2^_g,CV_, or *r*^2^_i,CV_) than those with shorter distances (Supplementary Fig. S9). This is consistent with findings in diverse panels of rice (*Oryza sativa*) and maize (*Zea mays*) that within-subpopulation genetic variance dominated predictions (Guo et al. 2014), but inconsistent with findings in Barley (*Hordeum vulgare* L.) that GP accuracy decreased with the increase of genetic distances in the training population (Lorenz and Smith 2015). Taken together, these results indicate that read depth for genetic variants and population structure are potentially another 2 factors influencing GP accuracy, especially for octoploids in switchgrass.

### Insights of molecular mechanisms underlying trait determination by interpreting GP models

Besides the prediction of complex traits, the GP models can be used to get insights of molecular mechanisms underlying trait variations as well, by examining the absolute coefficients of genetic variants in the trained models. Variants with absolute coefficients ranked above 95th or 99th percentiles were considered as important to trait prediction, and genes harboring or nearby the important variants (<3.5 kb) were considered as important genes. We focused on the anthesis date models since the current knowledge for genetic bases of flowering time is the most abundant among all the 20 traits studied here, and anthesis date had the highest *r*^2^_i,CV_ (Supplementary Fig. S4) and one of the highest *r*^2^_i,test_ (Supplementary Fig. S7). To test how well anthesis date models recover flowering time genes, we collected 23 maize and 378 Arabidopsis flowering time genes (Supplementary Table S5) to identify putative orthologs in switchgrass that were used as benchmark flowering time genes (hereafter referred to as FT-genes; see Materials and methods and Supplementary Table S6).

**Figure 7.**
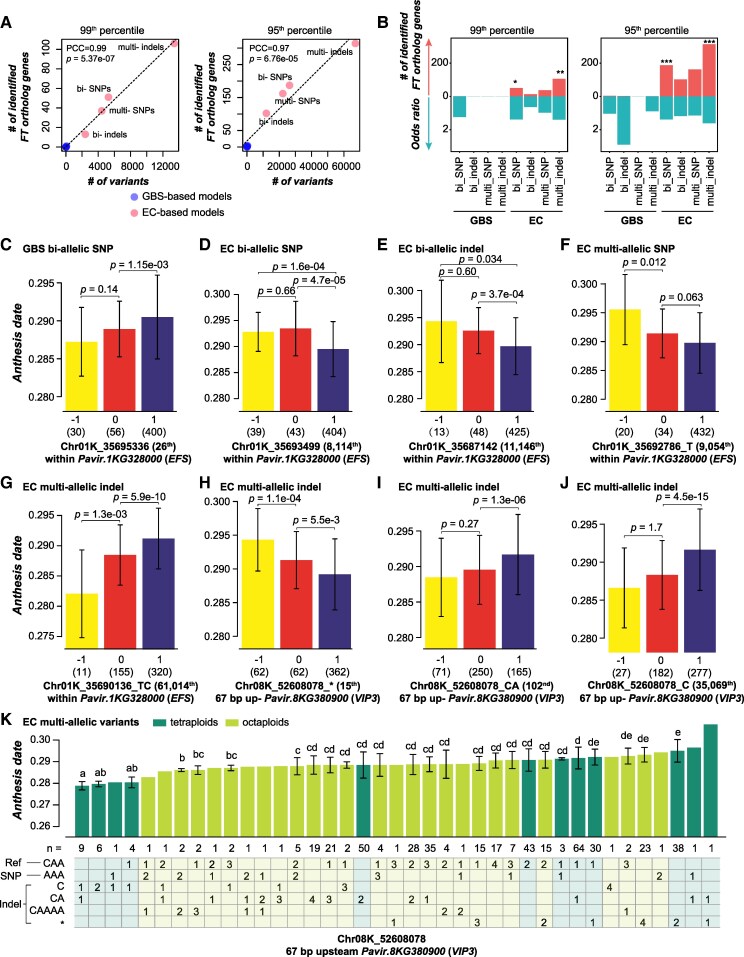
Analysis of important variants for genomic prediction models. **A)** Correlation between the numbers of variants with absolute coefficient above 99th (left panel) or 95th (right panel) percentile (*x* axis) and the corresponding numbers of switchgrass genes putatively orthologous to known flowering time genes (FT-genes, *y* axis). Dashed line: the diagonal line. **B)** The number of FT-genes identified by models and the corresponding odds ratio when 99th (left panel) and 95th (right panel) percentiles were used as cutoff thresholds. *P-value from Fisher's exact test < 0.05; **P-value < 0.01; ***P-value < 0.001. **C** to **J)** Anthesis dates of individuals with different alleles. The type of variants which were used to build the models is listed above the figures. The absolute coefficient rank of the variant is listed in the parenthesis on the right of the variant. *EFS*: *EARLY FLOWERING IN SHORT DAYS*; *VIP3*: *VERNALIZATION INDEPENDENCE 3*. Yellow, red, and blue bars in **C)** to **J)**: individuals with homologous alternative (−1), heterozygous (0), and homologous reference (1) alleles, respectively. *P*-values were from Wilcoxon signed-rank test; error bar: standard deviation with sample sizes indicated in parentheses. In **K)**, 6 types of alleles for the locus Chr08K_52608078 are indicated on the left of the table; values in the table indicate the numbers of the corresponding alleles; error bar: standard deviation with sample sizes underneath the bars. Only columns with >1 individual are indicated with letters indicating the statistical significance from Wilcoxon signed-rank test; columns sharing no same letters are significantly (*P* < 0.05) different from each other.

We found that the number of FT-genes identified based on absolute coefficients of variants in the models was not correlated with model performance (*r*^2^_i,CV_), either at the 95th (PCC = 0.56, *P*-value = 0.15) or 99th percentile (PCC = 0.45, *P*-value = 0.27) threshold, suggesting that models with better anthesis date prediction did not necessarily have more FT-genes identified as important. In contrast, the number of FT-genes identified by a model was positively correlated with the number of variants used in the model (Fig. 7A; Supplementary Fig. S10 and Tables S7 to S15). Specifically, GBS bi-allelic SNPs, bi-allelic indels, multi-allelic SNPs, and multi-allelic indels identified only 4 (1), 2 (0), 0 (0), and 1 (0) FT-genes as important when 95th (99th) percentile was used as a threshold, respectively, whereas EC bi-allelic SNPs, bi-allelic indels, multi-allelic SNPs, and multi-allelic indels identified 187 (51), 102 (13), 162 (37), and 314 (106) FT-genes as important, respectively (Fig. 7B; Supplementary Table S15). Only models built using EC bi-allelic SNPs and EC multi-allelic indels identified significantly more FT-genes than random guessing (*P*-value for Fisher's exact test < 0.034; red bars in Fig. 7B) and had the highest odds ratios among other models.

Out of the 7 FT-genes identified by GBS variants as important genes when the 95th percentile was used as the threshold, 6 were also identified by EC variants (Supplementary Fig. S10 and Table S15). For example, the GBS bi-allelic SNP Chr01K_35695336, which ranked 26th in terms of absolute coefficient, was located within the genic region of *Pavir.1KG328000*, a putative ortholog of Arabidopsis *EARLY FLOWERING IN SHORT DAYS* (*EFS*), which represses flowering in the autonomous promotion pathway (Soppe et al. 1999). Within *Pavir.1KG328000*, there were also several EC bi-allelic SNPs (e.g. Chr01K_35693499), bi-allelic indels (Chr01K_35687142), multi-allelic SNPs (Chr01K_35692786_T), and multi-allelic indels (Chr01K_35690136_TC) with absolute coefficients ranking above 95th percentile in the corresponding models. Individuals with homozygous reference alleles (1) for these loci flowered significantly differently as individuals with homozygous alternative (−1) and heterozygous alleles (0) (Fig. 7, C to G). Besides the 7 FT-genes identified by GBS variants, all the other identified FT-genes were EC variant-specific, further suggesting that EC variants are superior to GBS variants in either trait prediction or identification of potential contributing genes for traits in question.

Furthermore, we investigated in detail how multi-allelic variants contributed to trait prediction, by taking the locus Chr08K_52608078 as an example. This locus was located 67 bp upstream of *Pavir.8KG380900*, a putative ortholog of the Arabidopsis flowering time gene *VERNALIZATION INDEPENDENCE 3* (*VIP3*), which functions as an activator of the flowering-repressor gene *FLOWERING LOCUS C* (*FLC*) (Zhang et al. 2003). This locus had 6 alleles in the EC data among individuals studied here: the reference allele is CAA, and 5 alternative alleles included 1 SNP: AAA, and 4 indels: C, CA, CAAAA, and * (CAA was completely absent). In the model built using EC multi-allelic indels, Chr08K_52608078_* (ranked 15th; Fig. 7H), Chr08K_52608078_CA (ranked 102nd; Fig. 7I), and Chr08K_52608078_C (ranked 35,069th; Fig. 7J) had absolute coefficients above the 95th percentile, and individuals with different alleles of these 3 indels had significantly different anthesis date. Interestingly, when examining different alternative alleles for this single locus, individuals with homozygous reference alleles (see Materials and methods) flowered significantly earlier (Fig. 7H) or later (Fig. 7, I and J) than individuals with heterozygous alleles and homozygous alternative alleles, suggestive of the complexity of multi-allelic variants in trait determination. In addition, when examining the original allele compositions of this locus among individuals (Fig. 7K), we found that there were 39 combinations of all the 6 alleles. Tetraploid individuals with homozygous or heterozygous alleles of C flowered earliest, and tetraploid individuals with */*, AAA/CA, and CA/* flowered latest, whereas all the other tetraploids and all the octoploids flowered medially. This finding suggests that a C at this locus in the promoter sequence for 1 allele of Pavir.8KG380900 may interrupt the expression of this gene in tetraploids, leading to decreased expression of putative orthologs of *FLC* and resulting earlier flowering phenotypes, if the regulatory rules of *VIP3* and *FLC*-like genes in flowering are conserved between Arabidopsis and switchgrass. In contrast, this association between allelic C and flowering time was not clear for octoploids, suggesting a potentially more complex determination patterning of allelic components on gene functions (e.g. allele dosage), thus complex traits, in octoploids than in tetraploids. This type of analysis highlights the valuable insights into trait prediction provided by multi-allelic variants that were normally neglected by previous studies and the need to include multi-allelic variants in future studies of GWAS and GP.

## Discussion

Using switchgrass as a model system, our study showed the differences of 2 types of variants (e.g. GBS vs EC SNPs) for a given factor (e.g. genotyping approach) in the prediction accuracy for 20 traits and proposed suggestions that can be potentially taken in future GP practices. Generally, different versions of genome assemblies (short read-based vs. long read-based) had similar GP accuracy; genotyping approaches that capture variants with higher gene coverages should be considered with limited budgets; multi-allelic variants should be included in GP practices both for improving GP accuracy and identifying potential contributing variants for the target traits; and different types of variants should be integrated to improve the GP accuracy.

Although these conclusions summarized above were generally true for most traits, there were a few exceptions. For example, EC-based models tended to outperform GBS-based models for most traits, but the plant height (base of the longest flowering stem to the node at the base of the panicle) and total plant height (base of the longest flowering stem to the tip of the panicle) were predicted with consistently higher accuracy by GBS-based models, no matter whether the balanced, unbalanced, genic, or intergenic variants were used (Fig. 3A). This finding indicates that variations in plant height might be determined more by variants in genomic elements that are located within intergenic regions than those within genic regions. In addition, models built for tetraploids tended to have better prediction accuracy for most traits than those for octoploids, but the 2 plant height traits and ethanol (ethanol/g dry forage) were better predicted in octoploid models, both when GBS and EC variants were used (Fig. 6A). These exceptions, together with the findings in Wang et al. (2023b) that whether deep learning approaches outperformed classical approaches depends on the data sets used and traits in question, suggest that the optimal GP practices should be considered with caution on a case-by-case basis.

We found that related traits (i.e. anthesis date and heading date, plant height, and the total plant height) were generally predicted at a higher accuracy by the same data set regardless of the factor investigated (Figs. 2 to 4), but did not necessarily have highly correlated prediction accuracy across different models (e.g. plant height and total plant height; Fig. 5A). In another paper of ours (Wang et al. 2024), 2 flowering time-related traits (i.e. flowering time and rosette leaf number) had close but not highly correlated prediction accuracy across different models; models built for these 2 flowering time-related traits identified different sets of known flowering time genes. These findings suggest that the methods used for measuring morphological traits also impact GP accuracy and the identification of potentially important genes and should be carefully selected to ensure they are the most appropriate for the traits in question.

Based on our findings, in switchgrass, short read-based assembly led to comparable (for 12 traits) or even significantly better prediction accuracy (for 7 traits) for complex traits, whereas long read-based assembly only led to better prediction accuracy for anthesis date, for which the difference in *r*^2^_i,CV_ between short read- and long read-based models was only 0.016. Those results suggest that, for a plant species or lineage with only reference genome assembly based on short reads, it might not be necessary to update the assembly using long reads if only for improving the GP accuracy. However, if both the short and long read-based assemblies are available, long read-based assembly will still be a better option since (ⅰ) the differences in *r*^2^_i,CV_ were relatively small (max = 0.032) and (ⅱ) long read-based assemblies tend to be more commonly used by the community and are much more competent than short read-based assembly for calling SVs, another genetic variants that were not studied in this study but have shown great power in GP and GWAS practices.

In addition, our findings suggested that multi-allelic variants outperformed bi-allelic ones in trait prediction and identification of potential important genes that contribute to the formation of traits in question. Multi-allelic tandem repeats (He et al. 2024) and multi-allelic copy number variations (Handsaker et al. 2015) have been shown to contribute to the rice agronomic traits and gene expression dosage variations in humans, respectively. Although ∼10% (Jiang et al. 2020) or even a higher proportion (Kang et al. 2023) of genetic variants are multi-allelic, previous studies generally used bi-allelic variants only. Besides the strategy we used in this study to encode the multi-allelic variants, the BCFtools offered a model recently to handle loci with multiple alternative alleles (Danecek et al. 2021), which would help us make the best of all the genetic variants in GWAS and GP practices.

Our results indicate that gene coverage by variants had a substantial effect on GP accuracy, which gives recommendations for the selection of genotyping approaches and designation of SNP chips in future studies. A pan-genome study of 69 *A. thaliana* accessions showed that 18% of the 32,986 gene families were private to a single accession (Lian et al. 2024), indicating potential missing heritability by using a single reference genome. However, in most previous studies and our study, genetic variants were generally called by referencing a single genome assembly. Recently, more and more graph-based pan-genomes have been constructed to capture the missing heritability in plant species (Zhou et al. 2022; Kang et al. 2023; Yan et al. 2023; Liu et al. 2024). For example, including SVs identified based on pan-genome has been shown to significantly improve the estimated heritability for grapevine (*Vitis vinifera* ssp. *vinifera* L.) traits (Liu et al. 2024). It is important to know whether including other hidden genomic variants (e.g. SNPs and indels) revealed by pan-genome would also help improve GP accuracy for plant traits, especially for traits enabling a small group of ecotypes/cultivars/individuals to adapt to particular environments or endowing them certain horticultural characteristics to be distinguished from others.

In this study, we showed that models built for tetraploids tended to have better prediction accuracy for most traits than those for octoploids, potentially due to relatively longer genetic distances among tetraploids. Another potential reason that cannot be ruled out is that the allele dosage in octoploids was much more complex than those in tetraploids, whereas we took the strategy of pseudo-diploid genotyping that grouped all heterozygotes (e.g. AT, ATTT, AATT, and AAAT) into a single class. Some studies treated heterozygotes in an allele-dosage way (Yadav et al. 2024) or under different effect assumptions (namely, additive, duplex dominant, and simplex dominant; Rosyara et al. 2016; Wilson et al. 2021) when dealing with variants for polyploids. Considering that it is unlikely that genes/loci with different allele compositions across the whole genome have a same effect pattern and that multiple ploidy levels may coexist in some crops (e.g. diploids, triploids, tetraploids. and aneuploids coexist in *Phalaenopsis* orchids), tools that can detect the potential effect pattern for each locus based on prior knowledge or integrate all the possible effect patterns of alleles, and handle with multiple levels of ploidies simultaneously, are needed for crop selective breeding. The prior knowledge can be that, under which assumption, the statistical significance between genetic variants and traits of individuals with different allele compositions is the highest.

## Materials and methods

### Genomic and phenomic data

Two versions of switchgrass (*P. virgatum* L.) genome assemblies (v1 and v5) were downloaded from Phytozome (https://phytozome.jgi.doe.gov/pz/portal.html). The GBS data were obtained from Lu et al. (2013), and the raw data were downloaded from the National Center for Biotechnology Information (NCBI) under the project PRJNA201059 (Supplementary Table S1). The GBS barcodes for individuals in each pooled GBS library were obtained from the description of each Sequence Read Archive sample in the NCBI website. The EC data were from Evans et al. (2015), and the raw data were downloaded from NCBI under the project PRJNA280418 (Supplementary Table S1). Phenotypic data were from the diversity panel which consisted of 540 individuals from 66 populations (Lipka et al. 2014), including 7 morphological and 13 biochemical traits (Supplementary Table S2). The description of how the 7 morphological traits were measured is listed in Table 1 (Lipka et al. 2014). After filtering out individuals with low quality of GBS data, ploidy of 6, and the ones with information missing from any of the 20 traits, we retained 486 individuals in this study (Supplementary Table S2), including 263 tetraploids and 223 octoploids.

**Table 1.** Description of how the 7 morphological traits were measured (modified from Lipka et al. (2014))

Traits	Description
Anthesis date (days)	Cumulative growing degree days when 50% of panicles have 50% open florets
Heading date (days)	Cumulative growing degree days when ≥50% of stems are 50% emerged
Plant height (cm)	Base of the longest flowering stem to the node at the base of the panicle
Total plant height (cm)	Base of the longest flowering stem to the tip of the panicle
Leaf width (mm)	Widest part of the leaf below flag
Leaf length (mm)	Base to tip length of the leaf below flag
Standability	0∼10 (prostrate∼upright)

### SNP and insertion–deletion (indel) calling

Reads from the GBS and EC data were trimmed using Trimmomatic (Bolger et al. 2014) with parameters: 2:30:10 LEADING:3 TRAILING:3 SLIDINGWINDOW:4:20 MINLEN:35. Trimmed reads with scores > 20 and longer than 35 bp were kept for the following analysis. GBS reads were mapped to both 2 assemblies using bwa/0.7.12.r1044 (Li 2013) with default parameter setting, while EC reads were only mapped to the v5 assembly. The alignment after mapping was sorted using picardTools/1.113 (http://broadinstitute.github.io/picard).

SNP calling was conducted using GATK/3.5.0 (De Auwera and O’Connor 2020). For EC data, duplication alignments, which may be amplified during library preparation progress, were marked using MarkDuplicates module. No treatment was processed to GBS alignments, because reads in GBS data always had the same start point (the same restriction size, *Ape*KI site) (Lu et al. 2013) and stop point. SNPs and indels were identified using HaplotypeCaller module, with parameters of -stand_call_conf 40 -stand_emit_conf 10 –max_alternate_alleles 8 -ploidy x, where x = 2 for tetraploids (since the reference genome was also a tetraploid) while 4 for octoploids. Base-quality recalibration was conducted using BaseRecalibrator and IndelRealigner for SNPs and indels, respectively. Then, the HaplotypeCaller module was used for the second run of SNP and indel calling with the same parameter setting, and the output files were saved at GVCF format. All GVCF files for individuals were merged together using the CombineGVCFs module. The resulting SNPs were filtered using VariantFiltration with parameters of “QD < 2.0 || MQ < 40.0 || FS > 60.0 || MQRankSum < –12.5 || ReadPosRankSum < –8.0 –clusterSize 3 –clusterWindowSize 10”, while the indels were filtered with “QD < 2.0 || FS > 200.0 || ReadPosRankSum < –20.0”. Finally, SNPs and indels with minor allele frequency > 0.05 and missing data < 20% were kept in the final list.

### Encoding of multi-allelic variants

Multi-allelic variants were encoded using the method proposed by Zhan et al. (2016) with modifications, where m columns were encoded for m alternative alleles (m ≥ 1). For example, for a tetra-allelic variant (e.g. the reference allele is A, and 3 alternative alleles are T, G, and AT), there would be 3 columns (Table 2): A/A, A/T, or T/T for the first alternative allele; A/A, A/G, or G/G for the second; and A/A, A/AT, or AT/AT for the third. The first 2 columns were used as multi-allelic SNPs, and the third column was used as a multi-allelic indel. After encoding the multi-allelic variants, there were 1,628 GBS multi-allelic SNPs, 2,454 GBS multi-allelic indels, 444,102 EC multi-allelic SNPs, and 1,341,337 EC multi-allelic indels.

**Table 2.** Encoding for multi-allelic variants with A as the reference allele and T, G, and AT as the alternative alleles

Individual ID	Sequence	Column_1 (T)	Column_2 (G)	Column_3 (AT)
ID_1	A/A	1 (A/A)	1 (A/A)	1 (A/A)
ID_2	A/T	0 (A/T)	1 (A/A)	1 (A/A)
ID_3	T/G	0 (A/T)	0 (A/G)	1 (A/A)
ID_4	G/AT	1 (A/A)	0 (A/G)	1 (A/AT)
ID_5	AT/AT	1 (A/A)	1 (A/A)	−1 (AT/AT)

### Imputation of missing data

For bi-allelic SNPs in tetraploids, the missing data were imputed using fastPHASE (Scheet and Stephens 2006). Variants called for octoploids were treated as those called for tetraploids. For example, a variant with homozygous reference allele AAAA and a variant with homozygous alternative allele TTTT were treated as AA and TT, respectively, while all 3 types of heterozygous alleles—ATTT, AATT and AAAT—were treated as AT. For the bi-allelic indels, the reference allele was converted to “R”, and the alternative allele was converted to “A”, and then the missing data were imputed using fastPHASE. The multi-allelic variants were first encoded to bi-allelic ones and then were imputed using fastPHASE.

### GP model

To build GP models, homozygous reference alleles and alternative alleles were encoded as 1 and −1, respectively, while heterozygous variants were encoded as 0. The R package “rrBLUP” (Endelman 2011) was used to build the GP models because it is one of the methods which have relatively higher prediction accuracy compared with others in general (Lipka et al. 2014; Azodi et al. 2019). For each trait, all the 486 individuals were first split into training (389, 80%) and test (97, 20%) sets using the stratified strategies to make sure that individuals in training and test sets had similar trait value distribution. The training set was then used to establish predictive models, using a 5-fold CV scheme: (i) all the training individuals were first split into 5 folds; (ii) individuals in 4 folds (referred to as training subset) were used to build models, while individuals in the other fold (referred to as validation subset) were used to evaluate the model performance; (iii) the second step was conducted 5 times to make sure each fold will be used as validation subset once; and (iv) the *r*^2^ of PCC between true and predicted trait values of individuals in all 5 folds was calculated and was referred to as *r*^2^_CV_. This 5-fold CV scheme was repeated 10 times, and the median *r*^2^_CV_ value of 10 replicate runs was calculated. During the model training, the coefficient of each variant was calculated for each CV step and was averaged across 5 CVs. Then, the absolute mean coefficient of a variant was used as the measure of importance for this variant in a model.

To balance the numbers of variants used between the 2 models (e.g. models built using GBS bi-allelic SNPs vs those using EC bi-allelic SNPs), the one with more variants (i.e. EC bi-allelic SNPs) was down sampled to the same number as the one with fewer variants (GBS bi-allelic SNPs). This random down-sampling was conducted 100 times, resulting in 100 down-sampled data. The median prediction accuracy across the 100 models building using these 100 down-sampled data was used to measure the model performance.

### Association of GBS bi-allelic SNPs between 2 assemblies

To assess which GBS bi-allelic SNPs were shared between v1 and v5 assemblies, 2 approaches were explored in this study. First, bi-allelic SNPs from 2 assemblies were associated by aligning sequences of these 2 assemblies using MUMmer/4.0.0beta2 (Kurtz et al. 2004). Sequences of the v1 chromosomes and contigs were aligned with those of v5 chromosomes and scaffolds, using the function “NUCmer” with the option of –maxmatch. The function “mummerplot” was used to output the coordinates and the sequence identities of matched regions. Only regions with sequence identity > 95% were kept for downstream analysis. For each pair of matched regions, if the v1 region contains a SNP, then search for SNPs on the corresponding v5 region. A v1 SNP was associated with a v5 SNP if sequences 20 bp upstream or downstream of the v1 SNP matched to those of the v5 SNP (Supplementary Table S4).

The second approach is to align the v1 SNP in question and its neighbor sequences (500 bp up- and 500 bp downstream) to the v5 genome sequence using BLASTN (Altschul et al. 1990). If the best matched v5 region for a v1 1001 bp region contains a SNP at the same relative coordinate as the v1 SNP, then the v1 SNP and the v5 SNP are associated.

By comparing results from these 2 approaches, out of 7,556 v1 bi-allelic SNPs, 1,502 (19.9%) were associated with v5 SNPs from both approaches and 3,777 (50.0%) and 4 (0.05%) were associated with v5 SNPs from only blastn and Mummer methods, respectively. The corresponding v5 sequences of 2,118 v1 SNPs (28%) were not identified as having SNPs. This may be because SNPs were filtered by applying a hard threshold on continuous scores (i.e. parameter setting during SNP calling); thus, SNPs with scores close to the threshold may be filtered out in one assembly but not in the other one. The flank regions of 5 v1 SNPs had no matched sequences in v5, 83 v1 SNPs had no matches in v5 assembly near the SNPs, and 67 had different alleles between v1 and v5 assemblies (Supplementary Table S4).

### Putative orthologs to known flowering time genes

Known genes involved in flowering time in maize (*Z. mays*) were gained from Azodi et al. (2020) and the Maize Genetics and Genomics Database (https://www.maizegdb.org/). Flowering time genes in *Arabidopsis thaliana* were gained from the Flowering Interactive Database (http://www.phytosystems.ulg.ac.be/florid/, Bouché et al. 2016). Putative orthologs in switchgrass to these known flowering time genes were identified using the software OrthoFinder (Emms and Kelly 2019) with the default settings. All the known flowering time genes and their putative orthologs in switchgrass are shown in Supplementary Tables S5 and S6. Genetic variants that were located in intergenic regions were associated with the nearby genes which are closest to the variants and with distances to the variants < 3.5 kb.

### Accession numbers

Sequence data from this article can be found in the Phytozome 13 data libraries under accession numbers *Pavir.1KG328000* (*EARLY FLOWERING IN SHORT DAYS*) and *Pavir.8KG380900* (*VERNALIZATION INDEPENDENCE 3*).

## Supplementary Material

kiaf188_Supplementary_Data

## Data Availability

All the scripts used in this study are available on GitHub at https://github.com/ShiuLab/Manuscript_Code/tree/master/2022_GP_in_Switchgrass.

## Supplementary data

The following materials are available in the online version of this article.

**Supplementary Figure S1.** Classification of different types of genetic variants.

**Supplementary Figure S2.** Prediction accuracy of models built using the population structure on the CV sets.

**Supplementary Figure S3.** Prediction accuracy of models built using genetic variants on the CV sets.

**Supplementary Figure S4.** Improvement of *r*^2^ of models built using different genetic variants on the CV sets.

**Supplementary Figure S5.** Prediction accuracy of models built using the population structure on the test sets.

**Supplementary Figure S6.** Prediction accuracy of models built using genetic variants on the test sets.

**Supplementary Figure S7.** Improvement of *r*^2^ of models built using different genetic variants on the test sets.

**Supplementary Figure S8.** Prediction accuracy of models built using different GBS variants and models integrating all GBS variants.

**Supplementary Figure S9.** Prediction accuracy of models built for 4 subpopulations.

**Supplementary Figure S10.** Putative flowering time orthologs associated with variants that had absolute coefficients above the 99th percentile in 8 models.

**Supplementary Table S1.** Genomic sequencing sample information.

**Supplementary Table S2.** Twenty trait values for 486 individuals.

**Supplementary Table S3.** Summary of SNP/indel information detected using GBS or EC sequencing.

**Supplementary Table S4.** Association between bi-allelic SNPs between 2 assemblies.

**Supplementary Table S5.** Benchmark flowering time genes in Arabidopsis and maize and their putative orthologs in switchgrass.

**Supplementary Table S6.** Putative ortholog groups for benchmark flowering time genes in 3 species.

**Supplementary Table S7.** Important variants above 95th (black) and 99th (red) percentiles for models built using GBS bi-allelic SNPs.

**Supplementary Table S8.** Important variants above 95th (black) and 99th (red) percentiles for models built using GBS bi-allelic indels.

**Supplementary Table S9.** Important variants above 95th (black) and 99th (red) percentiles for models built using GBS multi-allelic SNPs.

**Supplementary Table S10.** Important variants above 95th (black) and 99th (red) percentiles for models built using GBS multi-allelic indels.

**Supplementary Table S11.** Important variants above 95th (black) and 99th (red) percentiles for models built using exome bi-allelic SNPs.

**Supplementary Table S12.** Important variants above 95th (black) and 99th (red) percentiles for models built using exome bi-allelic indels.

**Supplementary Table S13.** Important variants above 95th (black) and 99th (red) percentiles for models built using exome multi-allelic SNPs.

**Supplementary Table S14.** Important variants above 95th (black) and 99th (red) percentiles for models built using exome multi-allelic indels.

**Supplementary Table S15.** Identified important variants that are associated with putative orthologs of known flowering time genes

## Dive Curated Terms

The following phenotypic, genotypic, and functional terms are of significance to the work described in this paper:

VIP3 Gramene: AT4G29830
VIP3 Araport: AT4G29830
