# Supplementary material for: Optimizing genomic prediction for complex traits via investigating multiple factors in switchgrass
Source: Plant Physiol. 2025 May 7;198(3):kiaf188. doi: 10.1093/plphys/kiaf188 (PMC12238539; doi:10.1093/plphys/kiaf188)

Fig. S1 Classification of different types of genetic variants. (**A**) Frequency of different types of variants called by mapping the GBS data to v1 assembly. (**B**) Frequency of different types of variants called by mapping the GBS data to v5 assembly. (**C**) Frequency of different types of variants called by mapping the EC data to v5 assembly.

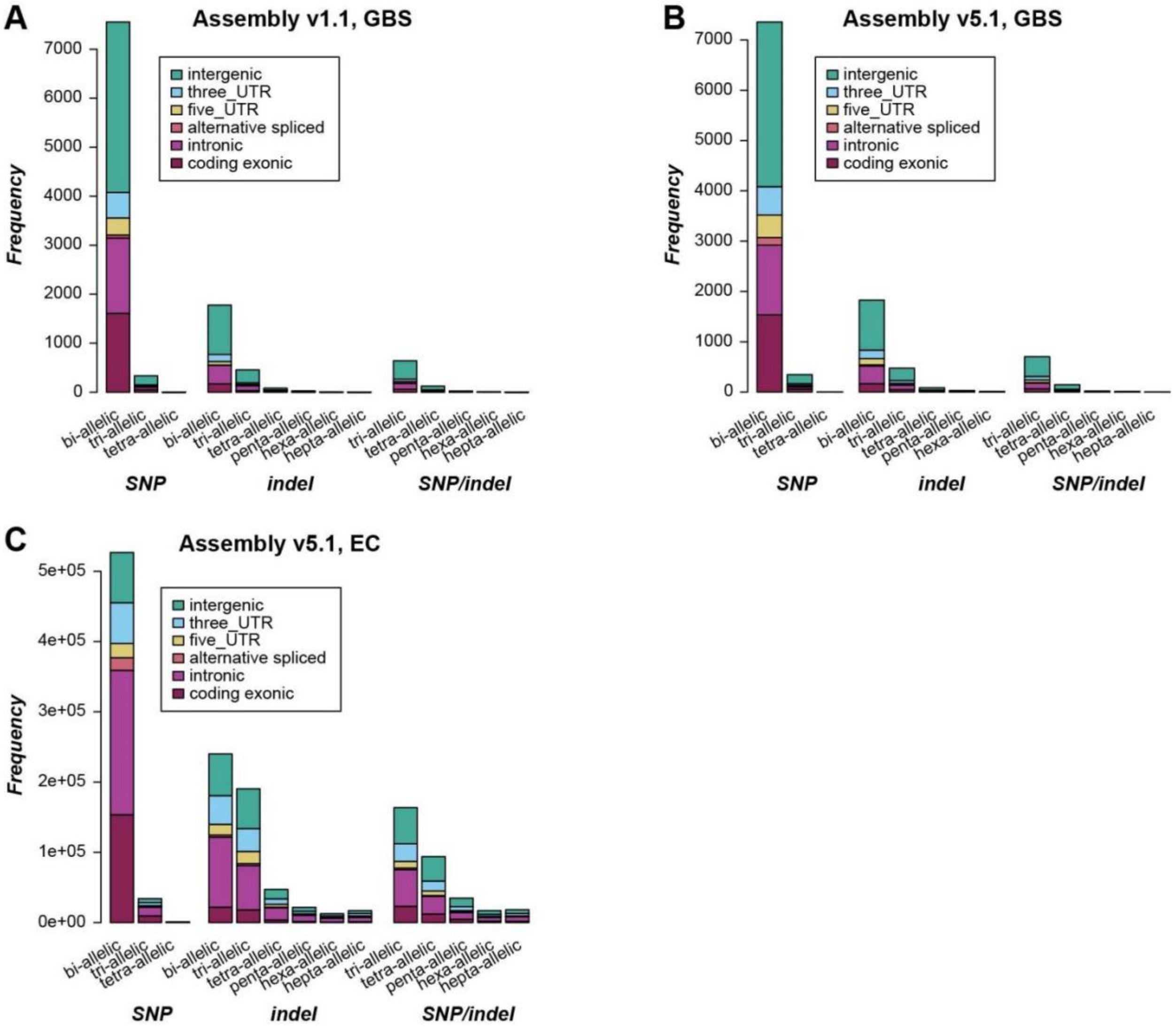

Fig. S2 Prediction accuracy of models built using the population structure on the cross-validation sets. (**A**) $r^2_{p,CV}$ for models built on both tetraploids and octoploids using different types of variants. Table above the heatmap shows different factors the variants used may belong to. For example, the 1st and 4th column show $r^2_{p,CV}$ of models built using bi-allelic SNPs called by mapping the GBS data to the v1 and v5 assembly, respectively; the 12th and 13th column show $r^2_{p,CV}$ of models built using balanced subset (down-sampled to the same number of GBS bi-allelic SNPs) and all the v5-based exome capture bi-allelic SNPs, respectively. (**B**) $r^2_{p,CV}$ for models built for different ploidy levels or subpopulations using GBS or exome capture bi-allelic SNPs. Color scale in the heatmap: median $r^2_{p,CV}$ among 10 replicate runs.

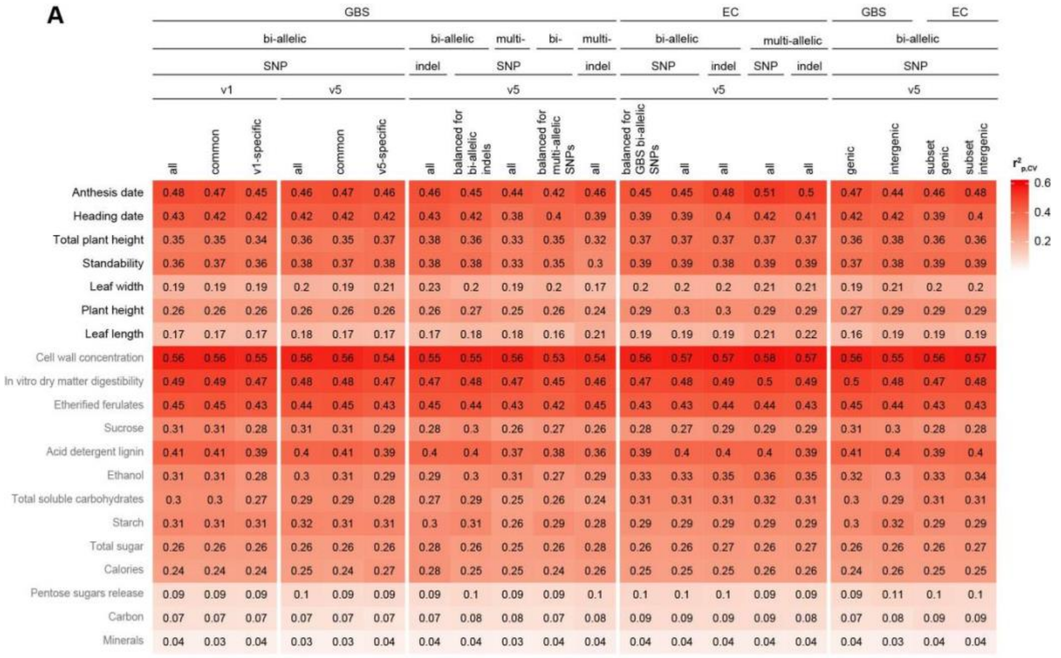

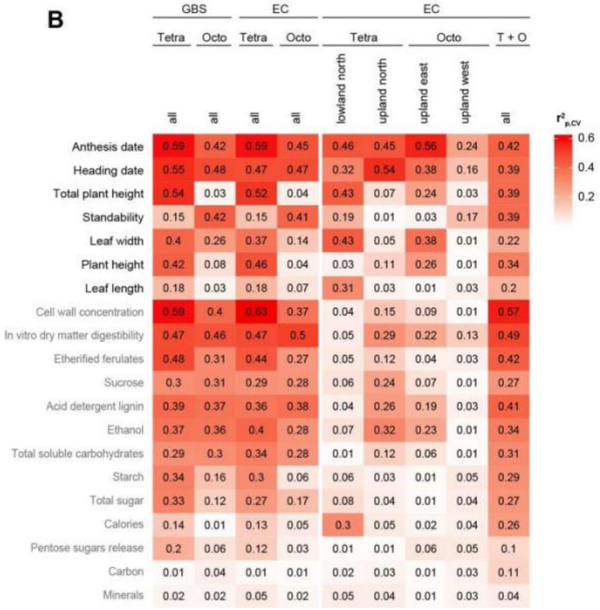

Fig. S3 Prediction accuracy of models built using genetic variants on the cross-validation sets. (**A**) $r^2_{g,CV}$ for models built on both tetraploids and octoploids using different types of variants. Table above the heatmap shows different factors the variants used may belong to. (**B**) $r^2_{g,CV}$ for models built for different ploidy levels or subpopulations using GBS or EC bi-allelic SNPs. Color scale in the heatmap: median $r^2_{g,CV}$ among 10 replicate runs.

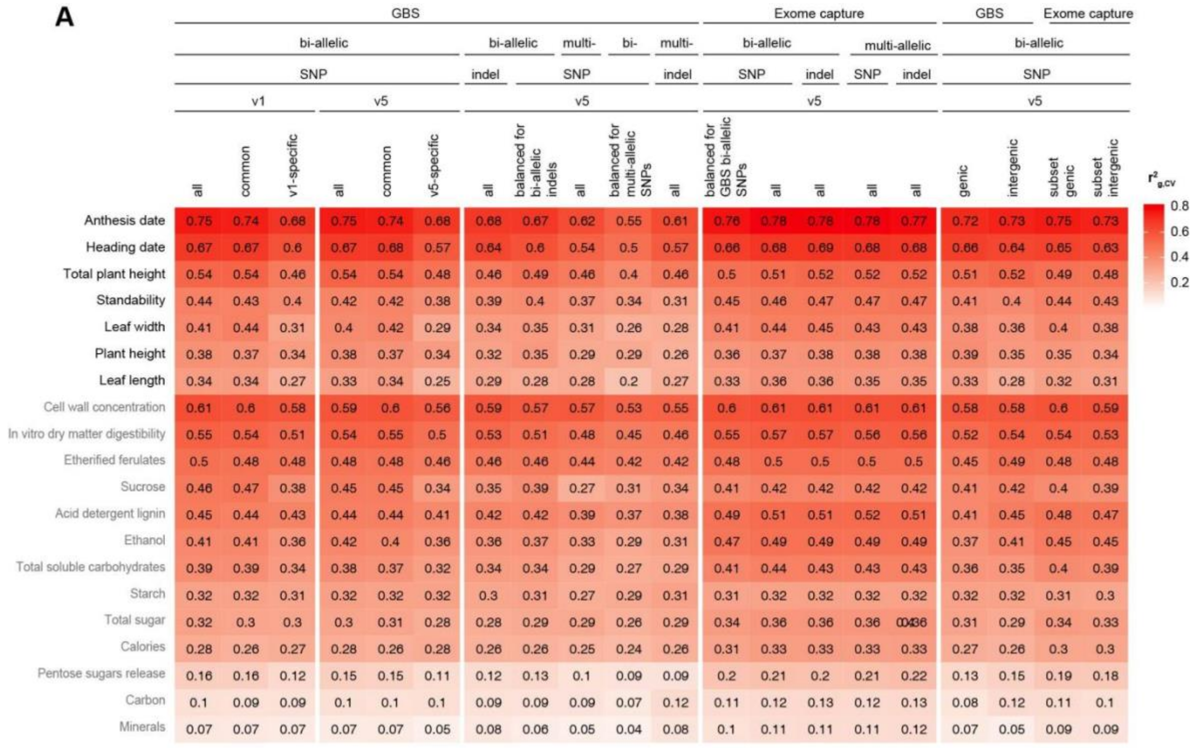

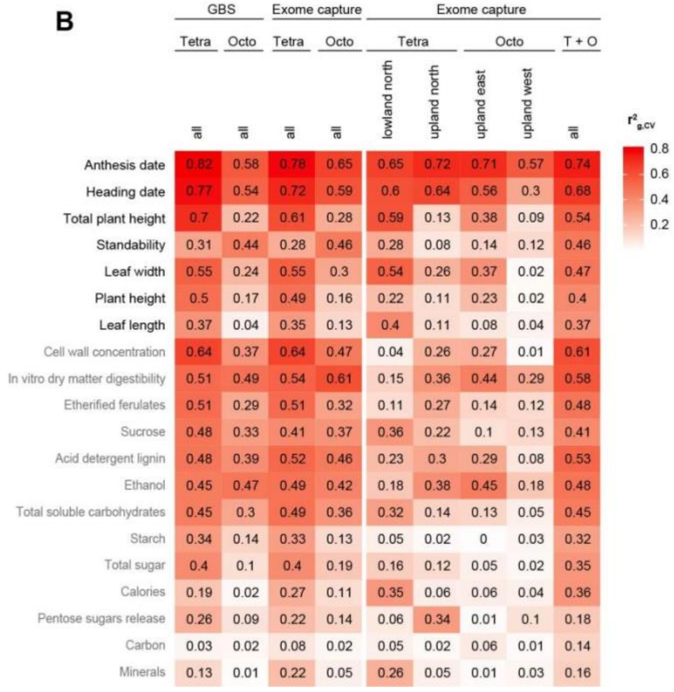

Fig. S4 Improvement of r^2 of models built using different genetic variants on the cross-validation sets. (**A**) $r^2_{i,CV}$ for models built on both tetraploids and octoploids using different types of variants. Table above the heatmap shows different factors the variants used may belong to. (**B**) $r^2_{i,CV}$ for models built for different ploidy levels or subpopulations using GBS or EC bi-allelic SNPs. Color scale in the heatmap: $r^2_{i,CV} \times 100$.

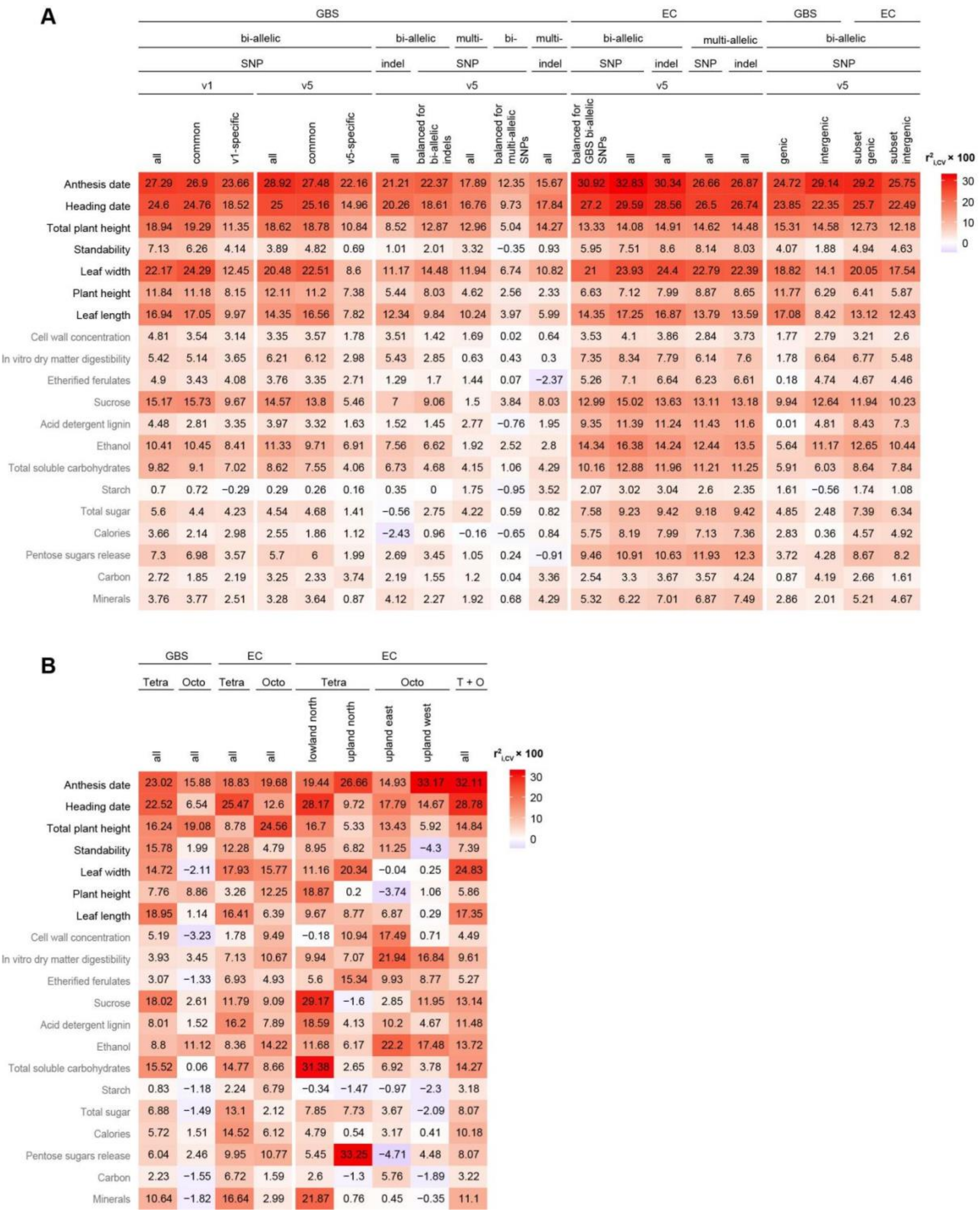

Fig. S5 Prediction accuracy of models built using the population structure on the test sets. (**A**) $r^2_{p,test}$ for models built on both tetraploids and octoploids using different types of variants. Table above the heatmap shows different factors the variants used may belong to. (**B**) $r^2_{p,test}$ for models built for different ploidy levels or subpopulations using GBS or EC bi-allelic SNPs. Color scale in the heatmap: median $r^2_{p,test}$ among 10 replicate runs.

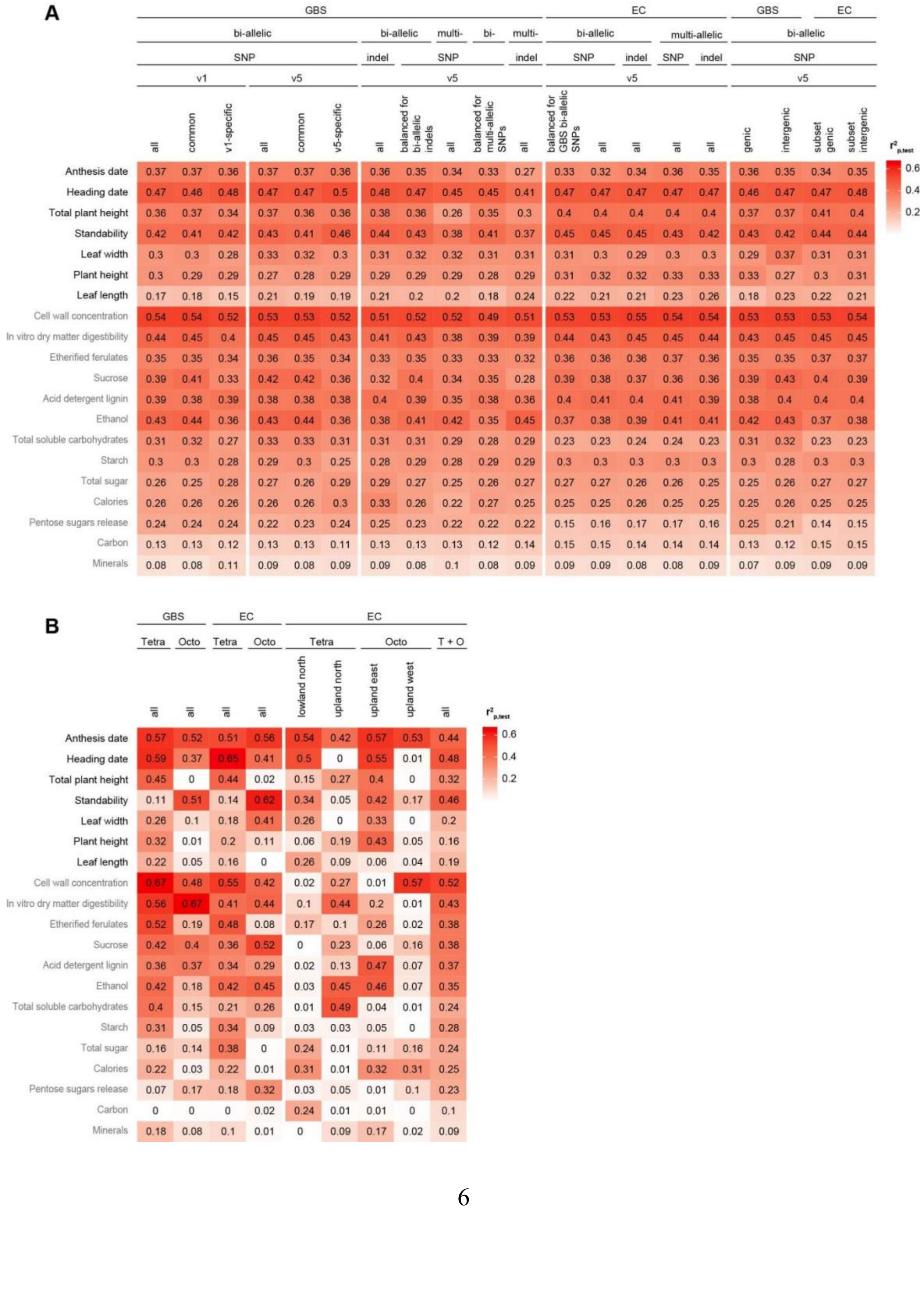

Fig. S6 Prediction accuracy of models built using genetic variants on the test sets. (**A**) $r^2_{g,test}$ for models built on both tetraploids and octoploids using different types of variants. Table above the heatmap shows different factors the variants used may belong to. (**B**) $r^2_{g,test}$ for models built for different ploidy levels or subpopulations using GBS or EC bi-allelic SNPs. Color scale in the heatmap: median $r^2_{g,test}$ among 10 replicate runs.

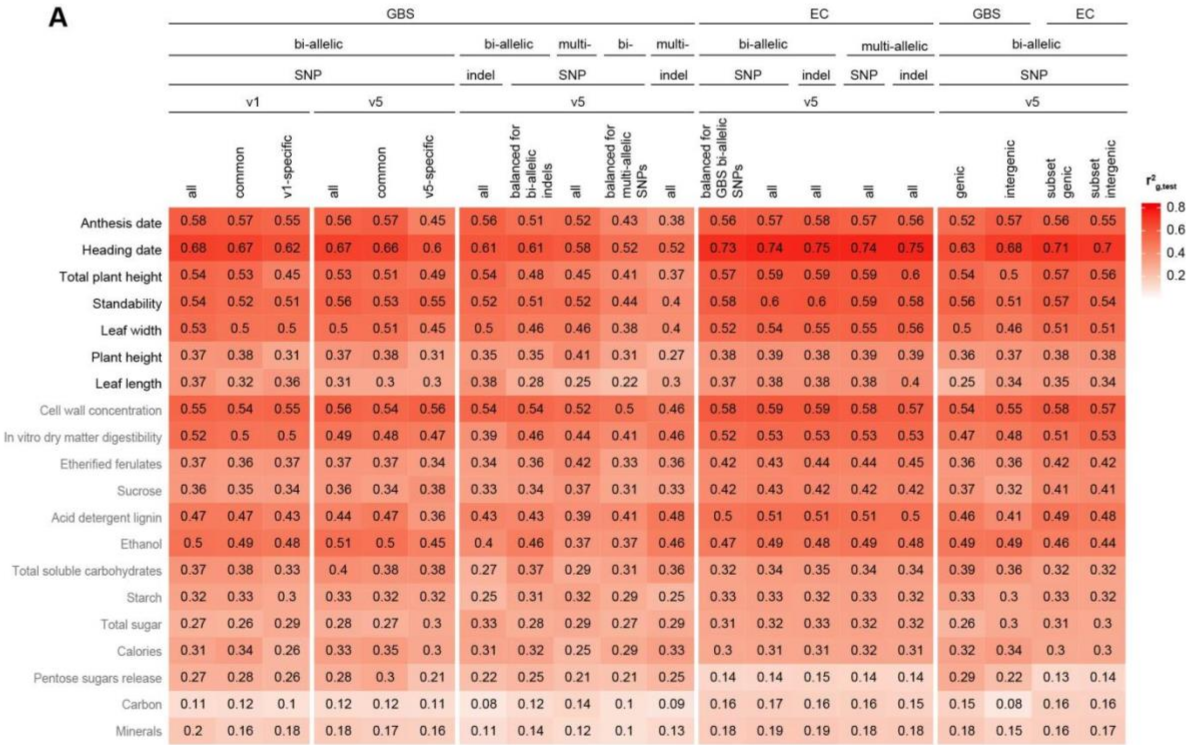

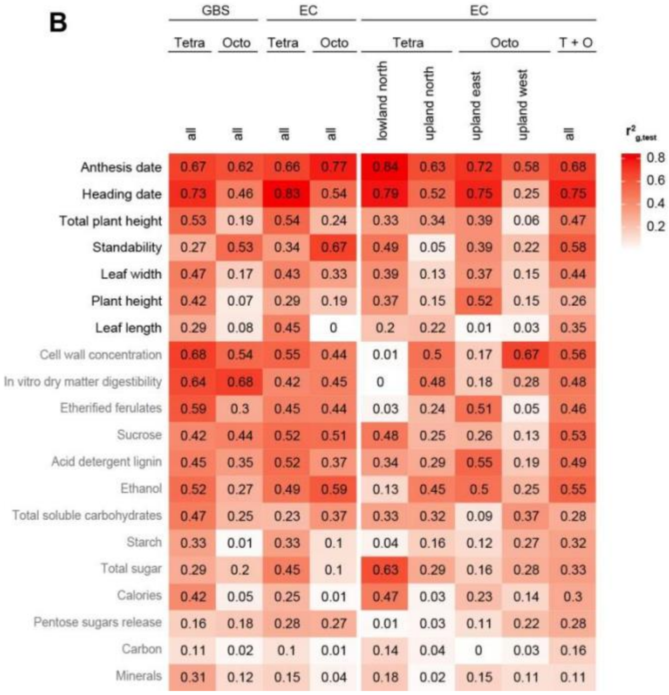

Fig. S7 Improvement of r^2 of models built using different genetic variants on the test sets. (**A**) $r^2_{i,test}$ for models built on both tetraploids and octoploids using different types of variants. Table above the heatmap shows different factors the variants used may belong to. (**B**) $r^2_{i,test}$ for models built for different ploidy levels or subpopulations using GBS or EC bi-allelic SNPs. Color scale in the heatmap: $r^2_{i,test} \times 100$.

A

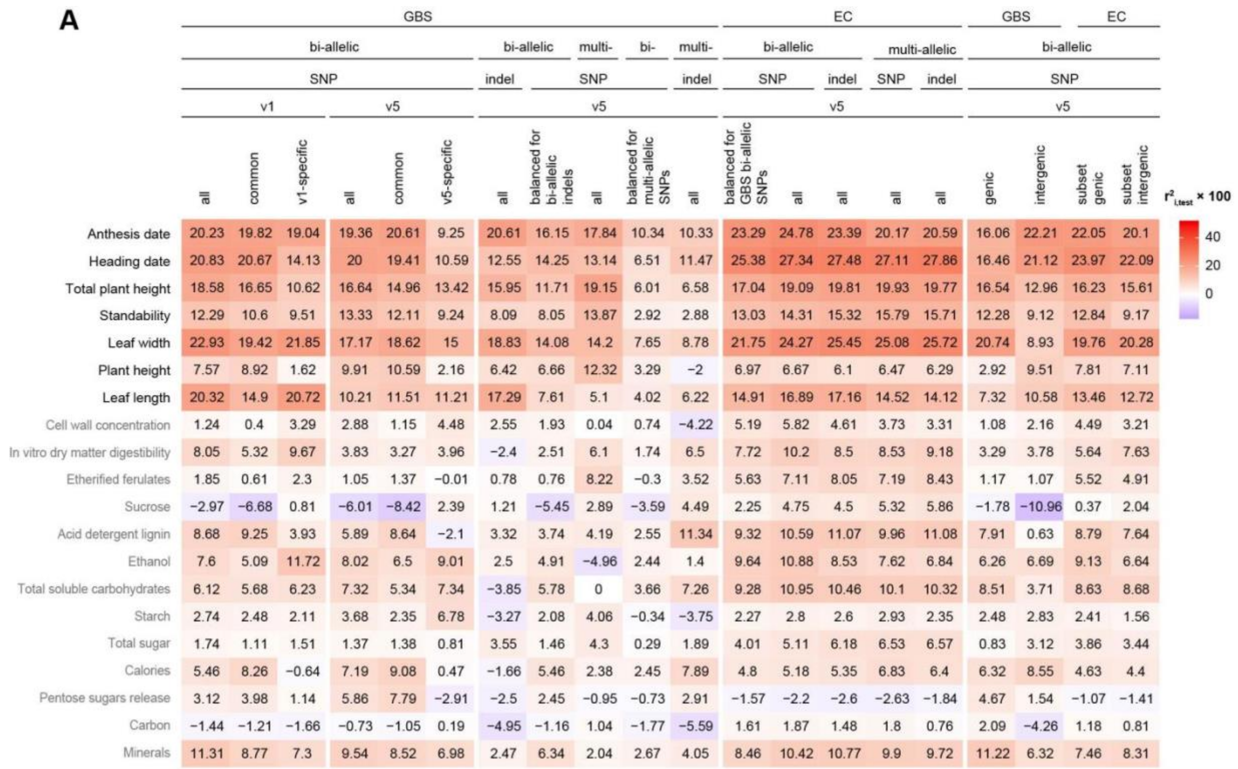

B

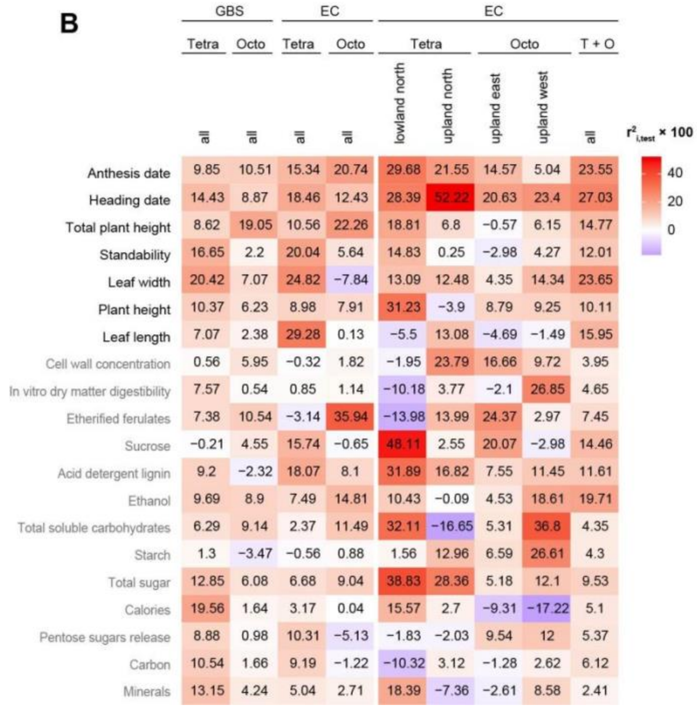

Fig. S8 Prediction accuracy of models built using different GBS variants and models integrating all GBS variants. Color and value in (**A-D**) indicate $r^2_{p,cv}$, $r^2_{p,test}$, $r^2_{g,cv}$, and $r^2_{g,test}$, respectively.

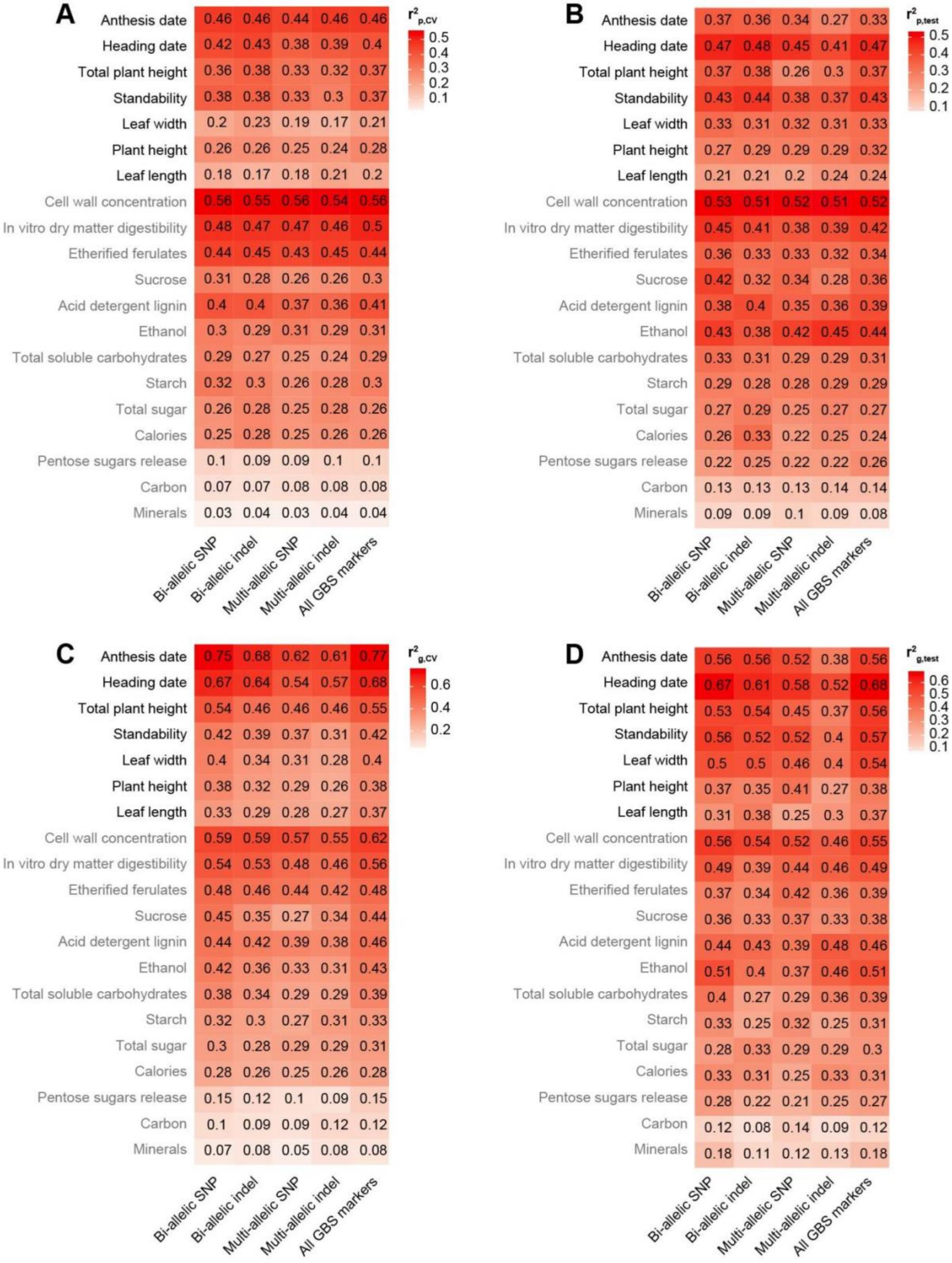

Fig. S9 Prediction accuracy of models built for four subpopulations. (**A-C**) The $r^2_{p,CV}$ (**A**), $r^2_{g,CV}$ (**B**) and $r^2_{i,CV}$ (**C**) for models using EC bi-allelic SNPs, built for Lowland north (4X) (dark blue), Upland north (4X) (blue), Upland east (8X) (green) and Upland west (8X) (yellow) subpopulations. The color background indicates that the model built for the corresponding subpopulation had the highest prediction accuracy for the trait in question. Error bar: standard deviation of 10 replicate runs.

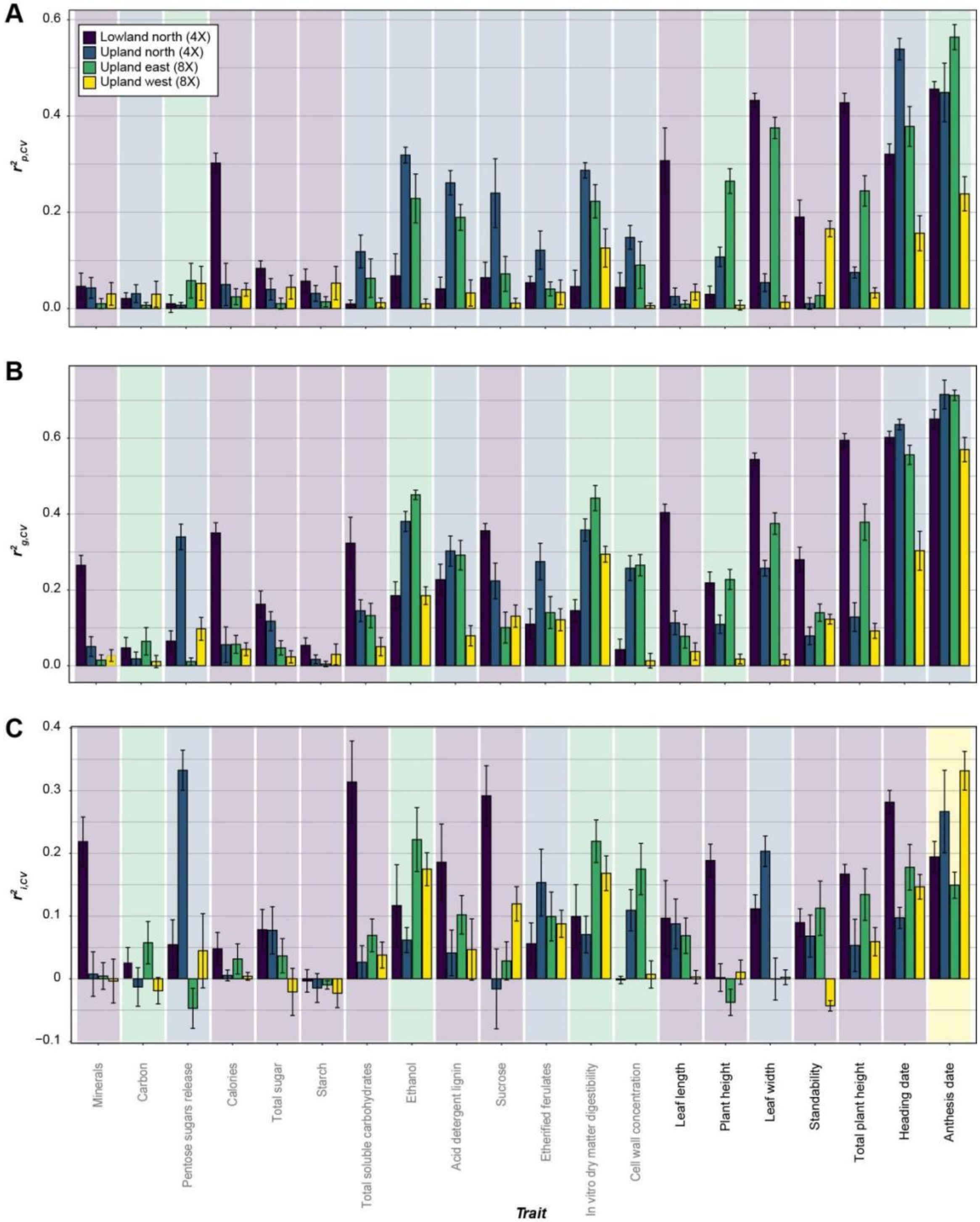

Fig. S10 Putative flowering time orthologs associated with variants that had absolute coefficients above the 99th percentile in eight models. The type of genetic variants used to build the models were listed above the heatmap. Yellow, green and blue: variants with absolute coefficients >99th, >95th and ≤95th percentiles, respectively.

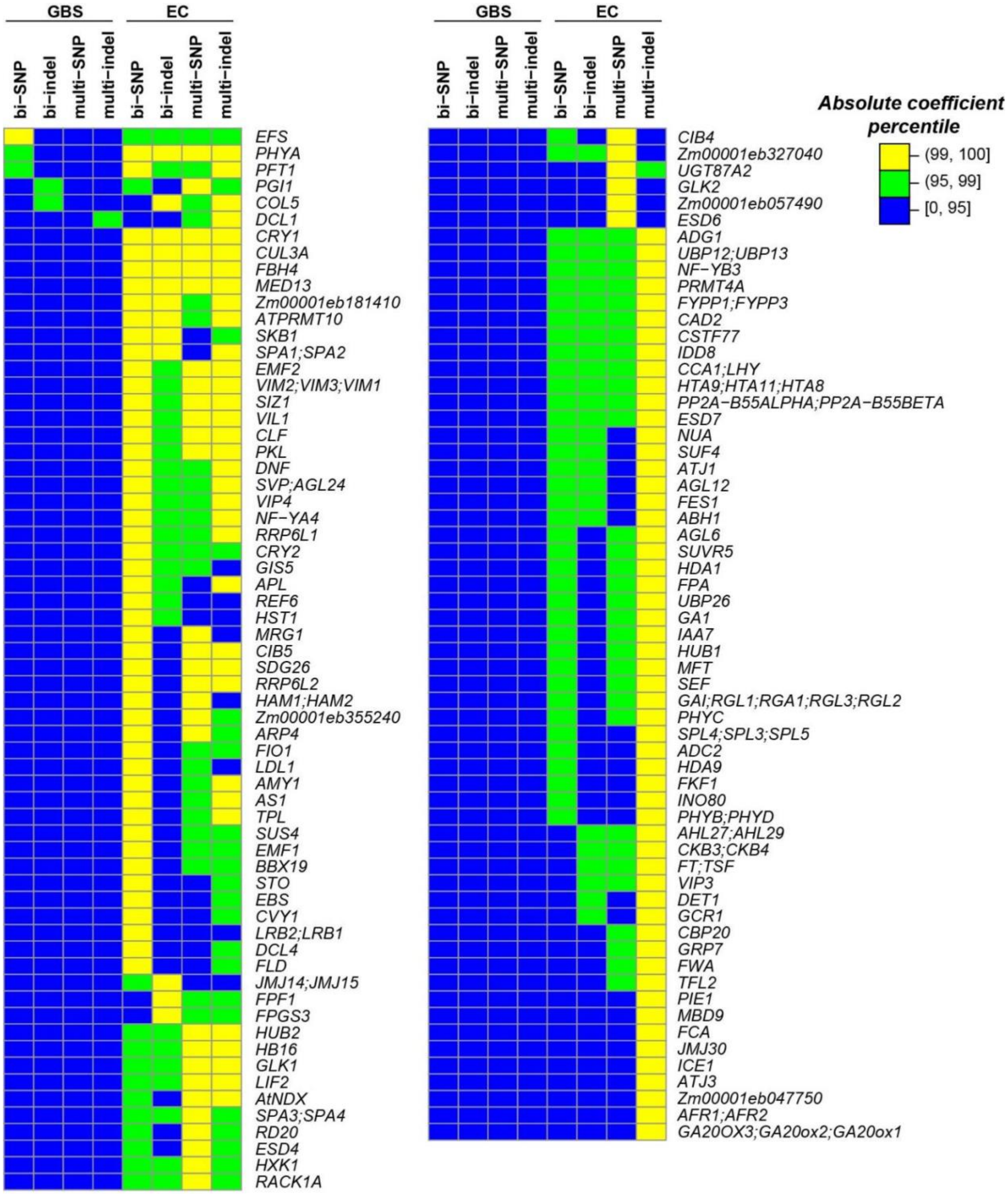

Supplement: kiaf188_Supplementary_Data [file kiaf188_supplementary_data.zip › PLPHYS-2025-0076R1_Supplementary Figures.pdf]
